# Tau phosphorylation impedes functionality of protective tau envelopes

**DOI:** 10.1038/s41589-025-02122-9

**Published:** 2026-01-27

**Authors:** Valerie Siahaan, Romana Weissova, Adela Karhanova, Eva Lanska, María J. Ruiz-Estrada, Barbora Pukajová, Vojtěch Dostál, Veronique Henriot, Carsten Janke, Lenka Libusová, Marcus Braun, Martin Balastik, Zdenek Lansky

**Affiliations:** 1 Institute of Biotechnology, Czech Academy of Sciences, BIOCEV, Vestec, Czech Republic; 2 Department of Cell Biology, Faculty of Science, Charles University, Prague, Czech Republic; 3 Institut Curie, Université PSL, CNRS UMR3348, Orsay, France; 4 Université Paris-Saclay, CNRS UMR3348, Orsay, France; 5 Institute of Physiology, Czech Academy of Sciences, Prague, Czech Republic

**Keywords:** Post-translational modifications, Cell biology, Single-molecule biophysics, Microscopy

## Abstract

Tau is an axonal microtubule-associated protein. Tau interaction with microtubules is regulated by phosphorylation. Hyperphosphorylation of tau is implicated in microtubule destabilization related to neurodegenerative disorders. However, how tau phosphorylation leads to microtubule destabilization is unknown. Recently, it was shown that tau molecules on microtubules cooperatively assemble into cohesive layers termed envelopes. Tau envelopes protect microtubules against degradation by microtubule-severing enzymes, suggesting a functional link between envelopes and microtubule stability. Here we show that tau phosphorylation has deleterious effects on the microtubule-protective function of tau envelopes. Using reconstitution and live-cell experiments, we found that tau phosphorylation destabilizes tau envelopes and decreases their integrity, leading to reduced microtubule protection against microtubule-severing enzymes. Our data suggest that a perturbation of microtubule homeostasis linked to tau hyperphosphorylation in neurodegeneration can be explained by the disassembly and impaired functionality of the tau envelopes.

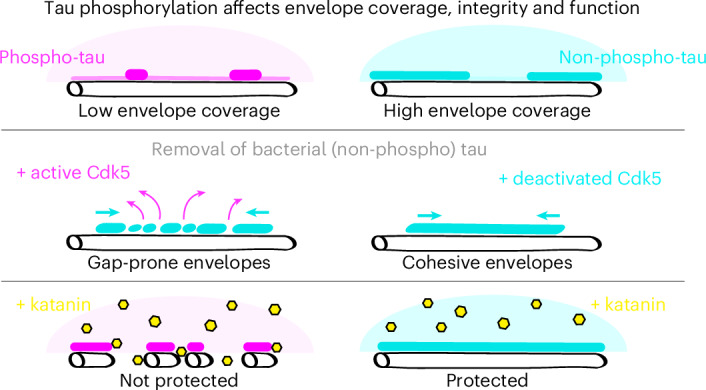

## Abstract (web summary)

Tau phosphorylation was found to hinder the formation and protective functionality of tau envelopes against microtubule-severing enzymes, providing a potential explanation for microtubule destabilization observed in neuropathology.

## Main

Microtubules are essential for neuronal function and homeostasis, for example, by providing tracks for intracellular cargo transport. The microtubule lifetime is regulated by a multitude of microtubule-associated proteins, which either affect microtubule assembly and disassembly^1,2^ or can sever microtubules into fragments^3^. Deregulation of various microtubule-associated proteins (for example, tau) can induce loss of microtubule mass from axons and dendrites and is associated with multiple neurodegenerative disorders^1,4^.

Tau is an intrinsically disordered microtubule-associated protein, which, in healthy neurons, localizes predominantly to axonal microtubules. Tau regulates the functioning of other microtubule-associated proteins and protects microtubules against microtubule-severing enzymes, such as katanin^5^. During neurodegeneration, tau is found aggregated in neurofibrillary tangles, which is one of the hallmarks of neurodegenerative disorders collectively termed tauopathies, such as Alzheimer disease^6,7^. It was proposed that tau aggregation causes a depletion of functional tau^8,9^, thereby leaving the axonal microtubules unprotected against microtubule-severing enzymes, such as katanin, which could lead to pathological microtubule destabilization. Importantly, aggregated tau has an increased phosphorylation state as compared to physiological tau and showed reduced interaction with microtubules^10,11^. These findings suggest a relationship between hyperphosphorylation of tau and microtubule instability related to neurodegeneration; nevertheless, the underlying molecular mechanism remains unclear.

It has recently been shown that tau molecules associate with microtubules in two distinct modes, either (1) diffusing individually along the microtubule lattice, rapidly binding and unbinding, or (2) binding cooperatively, with much longer interaction times, constituting cohesive envelopes also referred to as ‘condensates’ or ‘islands’ (refs. ^12–14^). These envelopes enclose the microtubules and act as selectively permeable barriers for other microtubule-associated proteins. Tau envelopes can differentially modulate the action of molecular motors by decreasing kinesin 1 walking distance^13,15^ while permitting dynein-mediated transport^12^. Moreover, microtubules covered by individually diffusing tau molecules are prone to disintegration by microtubule-severing enzymes, while tau envelopes efficiently protect the microtubule surface from katanin and spastin^12,13^. These observations suggest that the protective function of tau is mediated by the cohesion of tau envelopes. We, thus, hypothesized that the pathological effects of tau phosphorylation can be explained by the impact of tau phosphorylation on the stability and function of protective tau envelopes.

Here, we demonstrate that the formation and maintenance of tau envelopes are critically regulated by phosphorylation. We found that phosphorylation of tau decreases the propensity of tau to form envelopes and that envelopes formed by phosphorylated tau have altered functionality with decreased protection against microtubule-severing enzyme katanin. Our findings suggest that the cooperative binding mode of tau may provide a causal connection between tau phosphorylation and impaired tau functionality; the reduction of tau envelopes and their impaired functionality upon tau phosphorylation result in decreased microtubule protection from severing enzymes and, consequently, a decrease in microtubule stability.

## Results

### Tau phosphorylation induces envelope disassembly

**Fig. 1:**
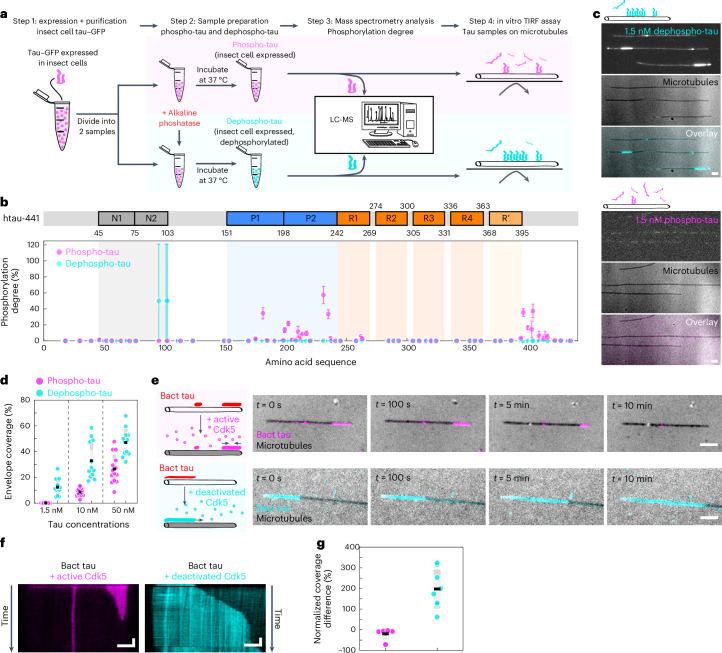
Tau phosphorylation induces envelope disassembly. **a**, Schematics of the sample preparation of phospho-tau (magenta) and dephospho-tau (cyan). **b**, MS-determined degree of phosphorylation of phospho-tau (magenta) and dephospho-tau (cyan). Data are presented as the mean ± s.d. (*n* = 3 replicates; Methods) and displayed at the location of the phosphorylation site along the amino acid sequence of tau (schematic of the sequence shown above the plot). Tau domains are color-coded: N-terminal domains (N1 and N2; gray), proline-rich domains (P1 and P2; blue), microtubule-binding repeats (R1–R4; orange) and domain pseudorepeat (R′, light orange). **c**, Multichannel fluorescence micrographs of taxol-stabilized microtubules (black; IRM) incubated with 1.5 nM dephospho-tau (cyan; top) or 1.5 nM phospho-tau (magenta; bottom). Scale bars, 2 μm. **d**, Percentage of taxol-stabilized microtubules covered with tau envelopes after 3 min of tau incubation. Data are presented as the mean ± s.d. (phospho-tau, magenta, *n* = 14 independent experiments; dephospho-tau, cyan, *n* = 12 independent experiments). **e**, Multichannel fluorescence micrographs of 15 nM Bact-tau (red in schematics) after addition of active Cdk5 (top; Bact-tau in magenta) or with heat-deactivated Cdk5 (bottom; Bact-tau in cyan). Microtubules (black) imaged using IRM. Scale bars, 2 μm. **f**, Time-projected kymograph corresponding to the micrographs from **e**. Scale bars, 2 μm (vertical) and 1 min (horizontal). **g**, Normalized difference between the tau envelope coverage before and 10 min after adding active Cdk5 (magenta; *n* = 6 independent experiments) or deactivated Cdk5 (cyan; *n* = 8 independent experiments), presented as the mean ± s.d. Source data

**Extended Data Fig. 1:**
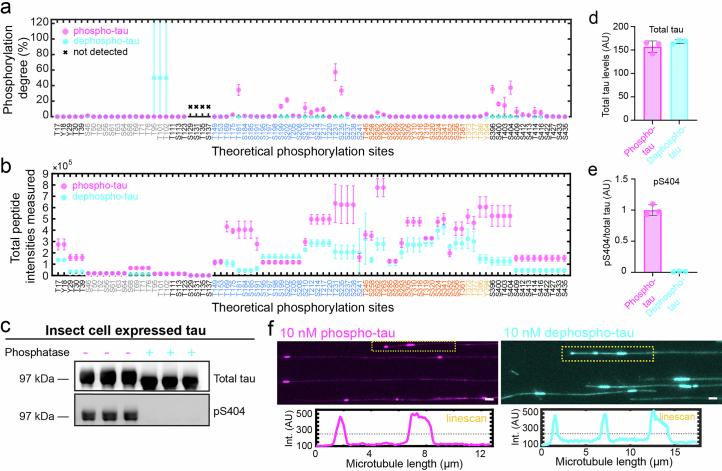
Tau phosphorylation reduces tau envelope propensity. **a**. Mass-spectrometry-determined degree of phosphorylation of phospho-tau (magenta) and dephospho-tau (cyan), presented as mean ± s.d. (n = 3, 3 independent samples). Phosphorylation sites with no peptide coverage are marked with a black cross. The color-coded legend corresponds to the colorscheme in Fig. 1b. **b**. Total relative intensities measured for all peptides covering a phosphorylation site, corresponding to data in panel **a** and Fig. 1b, presented as mean ± s.d. (n = 3, 3 independent samples). The color-coded legend corresponds to the colorscheme in Fig. 1b. **c**. Western blot of phospho-tau (insect cell expressed tau, magenta) and dephospho-tau (phosphatase-treated insect cell expressed tau, cyan) using total tau antibody (tau-5, top panel) and phospho-specific antibody (pS404, bottom panel). Three independent samples per condition were analyzed from one Western blot. **d**. Total tau levels (tau-5 intensity) of phospho-tau (magenta) and dephospho-tau (cyan) samples, presented as mean ± s.d. (n = 3, 3 independent samples). **e**. Quantification of relative phosphorylation levels, normalized to total tau. Mean phospho-tau (magenta) was set to 1 and compared dephospho-tau levels (cyan), presented as mean ± s.d. (n = 3, 3 independent samples). **f**. Fluorescence micrographs of 10 nM phospho-tau (left, phospho-tau in magenta) or 10 nM dephospho-tau (right, dephospho-tau in cyan) on taxol-stabilized microtubules. Linescans of the indicated microtubules (yellow dotted line) are shown below the micrograph. The grey dotted line indicates the half-maximum intensity threshold used to define tau envelope boundaries. Scale bars: 2 μm. Source data for **a**, **b**, **c**, **d**, **e** and **f** is available with this manuscript. Source data

To investigate whether phosphorylation of tau affects tau envelope formation, we expressed GFP-labeled human 2N4R tau (full-length tau protein, 441 aa), in insect cells and used alkaline phosphatase to dephosphorylate tau in vitro (Methods). This approach yielded two tau samples: phosphorylated tau (native, insect-cell-expressed tau, denoted as ‘phospho-tau’) and dephosphorylated tau (phosphatase-treated, insect-cell-expressed tau, denoted as ‘dephospho-tau’) (Fig. 1a). To confirm the efficiency of the phosphatase treatment, we determined the degree of phosphorylation using mass spectrometry (MS) (Methods and Fig. 1b; individual sites in Extended Data Fig. 1a,b) and western blot (Extended Data Fig. 1c–e). We then added the phospho-tau or dephospho-tau samples at 1.5 nM to surface-immobilized taxol-stabilized microtubules and visualized the interaction using total internal reflection fluorescence (TIRF) microscopy. While dephospho-tau readily formed micrometer-sized envelopes at this concentration, phospho-tau was present on microtubules only diffusively and did not form envelopes (Fig. 1c and Supplementary Videos 1 and 2). Repeating this experiment at two higher concentrations of tau showed that tau envelopes were formed by both tau samples (Extended Data Fig. 1f); nevertheless, at all concentrations tested, dephospho-tau covered a higher percentage of the microtubules (Fig. 1d), demonstrating an increased propensity of dephosphorylated tau to form envelopes.

**Extended Data Fig. 2:**
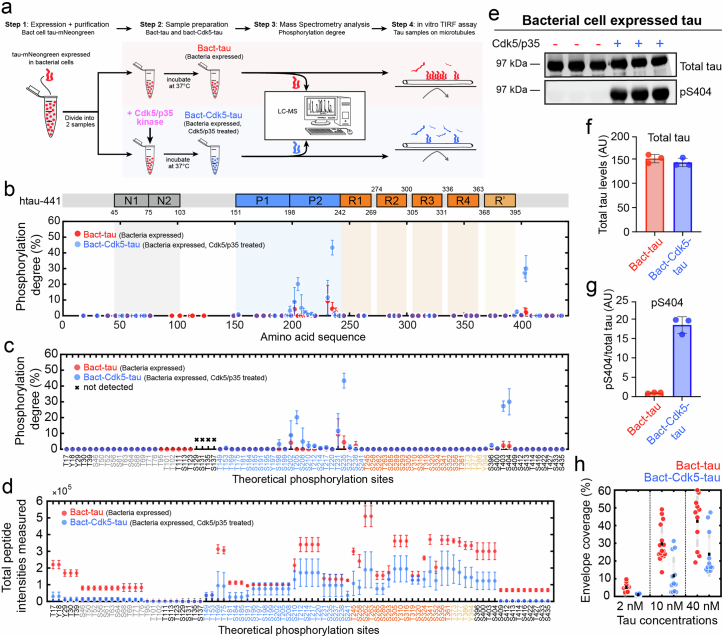
Cdk5 treatment increases tau phosphorylation. **a**. Schematics of sample preparation of bacterial expressed mNeongreen-tau (red), and Cdk5/p35-treated bacterial expressed mNeongreen-tau (blue). **b**. Mass-spectrometry-determined degree of phosphorylation of Bact-tau (red) or Bact-Cdk5-tau (blue) along the amino acid sequence (shown above the plot), presented as mean ± s.d. (n = 3, 3 independent samples). **c**. Mass-spectrometry-determined degree of phosphorylation of Bact-tau (red) or Bact-Cdk5-tau (blue) for individual phosphorylation sites, presented as mean ± s.d. (n = 3, 3 independent samples). Phosphorylation sites with no peptide coverage are marked with a black cross. **d**. Total relative intensities measured for all peptides covering the phosphorylation site, corresponding to data in panels **b** and **c**. Data is presented as mean ± s.d. (n = 3, 3 independent samples). **e**. Western blot of Bact-tau (bacterial cell expressed tau, red) and Bact-Cdk5-tau (Cdk5/p35-treated bacterial cell expressed tau, blue) using total tau antibody (tau-5, top panel) and phospho-specific antibody (pS404, bottom panel). Three independent samples per condition were analyzed from one Western blot. **f**. Total tau levels (tau-5 intensity) of Bact-tau (red) and Bact-Cdk5-tau samples (blue), presented as mean ± s.d. (n = 3, 3 independent samples). **g**. Quantification of relative phosphorylation levels, normalized to total tau. Mean Bact-tau (red) was set to 1 and compared Bact-Cdk5-tau levels (blue), presented as mean ± s.d. (n = 3, 3 independent samples). **h**. Percentage of taxol-stabilized microtubules covered with tau envelopes after 3 min incubation with Bact-tau (red, mean ± s.d., n = 12, 16, 11 independent experiments) or Bact-Cdk5-tau (blue, mean ± s.d., n = 12, 12, 11 independent experiments). Source data for **b**, **c**, **d**, **e**, **f**, **g** and **h** is available with this manuscript. Source data

To confirm our findings, we repeated these experiments with tau expressed in bacterial cells, which possesses a low phosphorylation state (denoted as ‘Bact-tau’) and increased its phosphorylation state using a kinase (Extended Data Fig. 2a and Methods). A multitude of kinases have been shown to phosphorylate tau, including proline-directed kinases (for example, cyclin-dependent kinase 5 (Cdk5), glycogen synthase kinase 3β or mitogen-activated protein kinase). Cdk5-mediated phosphorylation of tau has been shown to control multiple processes in neural development (for example, axonal growth and guidance), while hyperactivation of Cdk5 (for example, in Alzheimer disease) has been shown to result in heightened tau phosphorylation, promoting tau mislocalization, aggregation and formation of neurofibrillary tangles^8,16,17^. Therefore, we phosphorylated the Bact-tau sample using Cdk5 with its activator p35 (denoted as ‘Bact-Cdk5-tau’) (Extended Data Fig. 2a and Methods), yielding a sample with higher phosphorylation degree, as confirmed by MS (Extended Data Fig. 2b; individual sites in Extended Data Fig. 2c,d) and western blot (Extended Data Fig. 2e–g). We then added these samples separately to taxol-stabilized microtubules. In accordance with our previous results, we found that the Bact-tau formed larger envelopes at lower concentrations compared to Bact-Cdk5-tau (Extended Data Fig. 2h). Interestingly, when comparing the envelope coverage of 10 nM phospho-tau, which is phosphorylated at many different sites, to the envelope coverage of 10 nM Bact-Cdk5-tau, which is phosphorylated only at Cdk5-target sites, we found a similar envelope coverage (*P* = 0.32206), suggesting that Cdk5 nontarget sites might not be important for tau envelope formation.

We next asked whether tau phosphorylation can destabilize preexisting tau envelopes formed by dephosphorylated tau. To test this, we formed envelopes using 15 nM Bact-tau and, after 10 min, we added Cdk5 to the channel while keeping tau in solution. After the addition of Cdk5, we observed that the tau envelopes started to disassemble from their boundaries (Fig. 1e,f, Supplementary Video 3 and Methods), with occasional fission events within the envelope during disassembly (0.01 ± 0.08 fissions per mm per s). As a control, we repeated the experiment with heat-deactivated Cdk5 (Methods), in which case no disassembly was observed and, on the contrary, a significant increase in envelope coverage was detected (Fig. 1e–g and Supplementary Video 4). Combined, these experiments show that phosphorylation of tau decreases the propensity of tau to form envelopes and destabilizes preexisting envelopes.

### Phosphorylation reduces envelope integrity

**Fig. 2:**
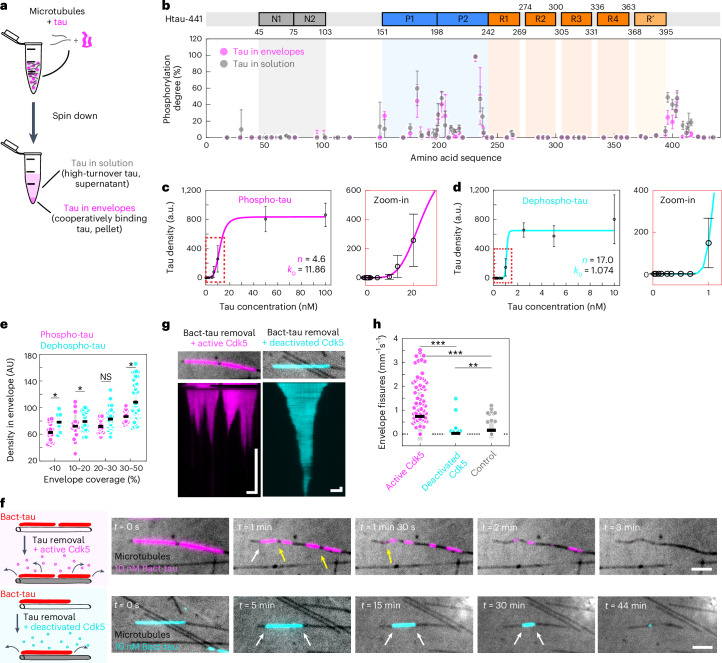
Phosphorylation reduces envelope integrity. **a**, Schematics of the sample preparation (Methods). **b**, MS-determined degree of phosphorylation of tau found in the pellet (slow-turnover tau in envelopes; magenta) and unbound tau in the supernatant (high-turnover tau in solution; gray), presented as the mean ± s.d. (*n* = 6 replicates; Methods) and displayed at the location of the phosphorylation site. **c**, Phospho-tau concentration plotted against density on the microtubules and fitted to the Hill–Langmuir equation (Methods) to obtain the Hill coefficient (*n* = 4.6) and dissociation constant (*k*_D_ = 11.86 nM). Data are presented as the mean ± s.d. (*n* = 2 independent experiments with *n* = 10, 20, 20, 20, 20, 25, 24, 21, 36, 31 or 24 acquisition areas containing at least five microtubules per area). A zoomed-in view of the low-concentration regime is shown next to the plot. **d**, Dehospho-tau concentration plotted against dephospho-tau density and fitted against the Hill–Langmuir equation to obtain the Hill coefficient (*n* = 17.0) and dissociation constant (*k*_D_ = 1.074 nM). Tau density is presented as the mean ± s.d. (*n* = 2 independent experiments with *n* = 16, 26, 27, 15, 27, 15, 23, 10, 30, 32, 24 or 27 acquisition areas). A zoomed-in view of the low-concentration regime is shown next to the plot. **e**, Density of tau within the envelope region for phospho-tau (magenta) and dephosho-tau (cyan) envelopes, presented as the mean ± s.d. (phospho-tau, *n* = 18, 16, 18 or 12 envelopes in five, three, four or one independent experiments, respectively; dephospho-tau, *n* = 7, 17, 30 or 37 envelopes in two, two, four or five independent experiments, respectively). Two-sided *t*-test *P* values (left to right): *P* = 0.0207, 0.2569, 0.0165 and 0.0169. **f**, Fluorescence micrographs of removal of 10 nM Bact-tau in the presence of active Cdk5 (top; Bact-tau in magenta) or heat-deactivated Cdk5 (bottom; Bact-tau in cyan). Microtubules (black) are visualized using IRM. Envelopes disassemble from the boundaries (white arrows) with occasional envelope fissure events (yellow arrows; only in the active Cdk5 example). Note the different timescales. Scale bars, 2 μm. **g**, Kymograph of the experiments shown in **f**. Fluorescence micrograph of the tau envelope at *t* = 0 min is depicted above the kymograph. Scale bars, 2 μm (horizontal) and 2 min (vertical). **h**, Number of fissure events within disassembling Bact-tau envelopes in the presence of active Cdk5 (magenta) or deactivated Cdk5 (cyan) or in absence of kinase (control; gray). Data are presented as the mean ± s.d. (*n* = 117, 119 or 52 envelopes in seven, eight or four independent experiments, respectively). Two-sided *t*-test *P* values (left to right): *P* = 8.85 × 10^−13^, 7.39 × 10^−5^ and 0.00136. *P < 0.05, **P < 0.01 and ***P < 0.001. Source data

**Extended Data Fig. 3:**
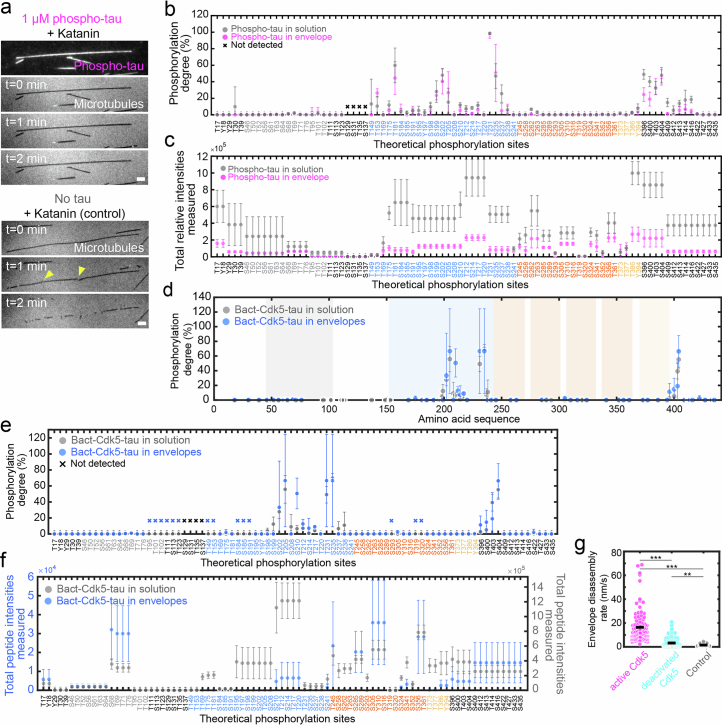
Phosphorylated tau participates in envelope formation. **a**. 100 nM Katanin-GFP was added to taxol-stabilized microtubules in presence of 1 µM phospho-tau (top panels), or in absence of tau (bottom panels). **b**. Mass-spectrometry-determined phosphorylation degree of spin down sample with phospho-tau in solution (grey) and phospho-tau in the pellet (phospho-tau in envelope, magenta), presented as mean ± s.d. (n = 6, 6 independent samples). Phosphorylation sites with no peptide coverage are marked with a black cross. The phosphorylation degree of tau in the pellet was slightly lower within the proline-rich region and C-terminal regions, and almost no phosphorylation was observed in the N-terminal region and microtubule-binding repeats for both tau samples. **c**. Total relative intensities of all peptides that covered a phosphorylation site, corresponding to data in panel **b** and Fig. 2b. Data is presented as mean ± s.d. (n = 6, 6 independent samples) for unbound tau from the supernatant (Bact-Cdk5-tau in solution, grey) and cooperatively bound tau from the pellet (tau in envelope, magenta). **d**. Mass-spectrometry-determined degree of phosphorylation of Bact-Cdk5-tau in solution (grey) or in envelopes (blue) along the amino-acid sequence of tau, presented as mean ± s.d. (n = 3, 3 independent samples). **e**. Mass-spectrometry-determined degree of phosphorylation of Bact-Cdk5-tau in solution (grey) or in envelopes (blue) for individual phosphorylation sites, presented as mean ± s.d. (n = 3, 3 independent samples). Phosphorylation sites with no peptide coverage are marked with a black cross. We detected a minor increase in phosphorylation degree within the proline-rich region and C-terminal region in tau found in solution. **f**. Total relative intensities measured for all peptides covering the phosphorylation site, corresponding to data in panels **d** and **e**, presented as mean ± s.d. (n = 3, 3 independent samples). **g**. Envelope disassembly rate after addition of active Cdk5 (magenta), deactivated Cdk5 (cyan), or in absence of Cdk5 (control, grey), presented as mean ± s.d., n = 99, 123, 38 envelopes in 7, 8, 4 independent experiments. Two-sided t-test p-values (left to right): p = 2.30*10-20, p = 5.92*10-10, p = 0.0075. Source data for **b**, **c**, **d**, **e**, **f** and **g** is available with this manuscript. Stars for statistical tests represent: ** p < 0.01, *** p < 0.001. Source data

We next asked why tau phosphorylation leads to a decrease in envelope formation. We hypothesized that phosphorylated tau might not be able to participate in the formation of tau envelopes. Our phosphorylated tau samples are heterogeneous, meaning that they consist of tau molecules with different patterns and degrees of phosphorylation, which could be differently competent in forming envelopes. To test which tau molecules, from a given sample, can participate in envelope formation, we added phospho-tau at high concentration (1 μM) to taxol-stabilized microtubules (100 nM) in solution. At this elevated concentration, tau forms envelopes along the entire microtubule lengths, appearing as a uniform density covering the full microtubule, which protects the microtubule against microtubule-severing enzyme katanin (Extended Data Fig. 3a), a characteristic specific to tau envelopes^13^. By spinning down the 1 μM tau-covered microtubules, we could then separate the sample into (1) tau, which participated in envelope formation (cooperatively bound tau in envelopes, found in the pellet along with the microtubules) and (2) tau, which did not participate in envelope formation (tau in solution, found in the supernatant) (Fig. 2a and Methods). We then asked whether the pelleted fraction consisting of tau participating in envelopes would have a lower phosphorylation state. Interestingly, we found no striking differences in the phosphorylation patterns between the two samples (Fig. 2b; individual sites in Extended Data Fig. 3b,c), demonstrating that phosphorylated tau participates and is competent in envelope formation. We then repeated this pelleting assay using bacterial-expressed tau that was phosphorylated with Cdk5/p35 (Bact-Cdk5-tau) and likewise found that the phosphorylation degree was comparable between tau that participated in envelope formation (found in the pellet) and tau not participating in envelope formation (found in the supernatant) (Extended Data Fig. 3d; individual sites in Extended Data Fig. 3e,f). Combined, these data demonstrate that, although phosphorylated tau has a lower propensity to form envelopes, tau phosphorylation does not fully prevent tau from binding to microtubules cooperatively and thereby participating in tau envelope formation.

Knowing that phosphorylated tau participates in envelope formation, we hypothesized that the lowered propensity to form envelopes is caused by a combination of (1) reduced affinity of phosphorylated tau to the microtubule, as observed previously^10,11,18–21^, and (2) reduced cooperativity of the tau–microtubule interaction. Titrating either phospho-tau or dephospho-tau to microtubules and calculating the dissociation constant and Hill coefficient indeed revealed that tau phosphorylation reduces both tau affinity to the microtubule and the cooperativity degree (Fig. 2c,d and Methods).

We next tested the consequences of the decrease in tau–microtubule affinity and cooperativity because of tau phosphorylation on the structure and function of the tau envelopes. We prepared tau envelopes of either phospho-tau or dephospho-tau on taxol-stabilized microtubules and studied the density of tau molecules within the enveloped regions. We found that, for any given envelope coverage, the density of tau within the envelope region is lower in envelopes prepared from phospho-tau (Fig. 2e), suggesting that envelopes formed by phosphorylated tau consist of a less dense and potentially more gap-prone structure. To test this hypothesis, we prepared tau envelopes on taxol-stabilized microtubules using 10 nM Bact-tau (low phosphorylation degree) and removed tau from solution to observe the disassembly of the envelopes. The removal of tau from solution was performed in the presence of Cdk5 or heat-deactivated Cdk5 or in the absence of a kinase (control) (Methods). In presence of Cdk5, we observed that the disassembly of the envelopes was faster compared to the conditions with deactivated Cdk5 or the control (Fig. 2f,g (note the different experimental timeframes) and Supplementary Videos 5 and 6). Interestingly, we observed an increase in the number of fission events within the envelopes in the presence of Cdk5 (Fig. 2h and Extended Data Fig. 3g). Combined, these data show that tau phosphorylation reduces the cooperativity of tau molecules binding to the microtubules, which results in a decrease in the cohesiveness and, thus, the integrity of the envelopes.

### Tau phosphorylation increases turnover of tau in cells

**Extended Data Fig. 4:**
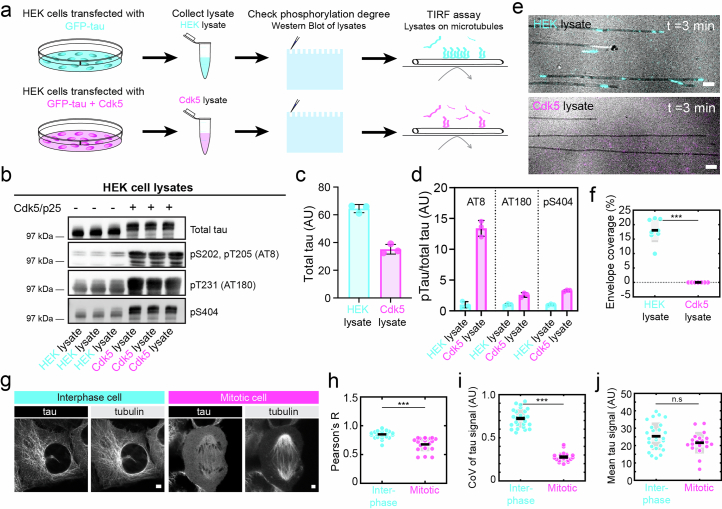
Tau phosphorylation reduces microtubule binding in cellular environment. **a**. Schematics of lysate preparation from HEK293T cells transfected with GFP-tau (HEK lysate, top, cyan) or GFP-tau and Cdk5/p25 (Cdk5 lysate, bottom, magenta). **b**. Western blot of HEK293T cells transfected with GFP-tau (HEK lysate) or GFP-tau and Cdk5/p25 (Cdk5 lysate) using total tau antibody (tau-5, top panel) and phospho-specific antibodies (AT8, AT180 and pS404). Three independent samples per condition were analyzed from one Western blot. **c**. Total tau levels (tau-5 intensity) of HEK (cyan) and Cdk5 lysate (magenta), presented as mean ± s.d. (n = 3, 3 independent samples). **d**. Quantification of relative phosphorylation levels, normalized to total tau. Mean HEK lysate (cyan) was set to 1 and compared to Cdk5 lysate levels (magenta), presented as mean ± s.d. (n = 3, 3 independent samples). **e**. Multichannel fluorescence micrographs of HEK (top, cyan) and Cdk5 lysate (bottom, magenta) on taxol-stabilized microtubules (black, IRM). Estimated tau concentrations: 30 and 40 nM, respectively. Scale bars: 2 μm. **f**. Percentage of microtubules covered by tau envelopes after addition of HEK (cyan) or Cdk5 lysate (magenta), presented as mean ± s.d. (n = 8, 8 independent experiments). Two-sided t-test, p = 4.3821*10-10. **g**. Fluorescence micrographs of GFP-tau (left) and mScarlet-tubulin (right) in U-2 OS cells in interphase (cyan) or in mitosis (magenta). **h**. Pearson’s R correlation coefficient between GFP-tau and mScarlet-tubulin signal, presented as mean ± s.d. (n = 25, 20 cells in 3, 3 independent experiments). Two-sided t-test, p = 1.4224*10-7. **i**. Coefficient of variation (CoV) of tau signal in the whole cell reflecting the difference between tau signal on microtubules and in cytoplasm. CoV for interphase (cyan) and mitotic (magenta) cells, presented as mean ± s.d. (n = 25, 19 cells in 3, 3 independent experiments). Two-sided t-test, p = 5.2528*10-20. **j**. Mean tau signal in interphase (cyan) and mitotic (magenta) cells as a measure of the expression level, presented as mean ± s.d. (n = 25, 19 cells in 3, 3 independent experiments). Two-sided t-test, p = 0.099936753. Source data for **b**, **c**, **d**, **f**, **h**, **i** and **j** is available with this manuscript. Stars for statistical tests represent: *** p < 0.001. Source data

To investigate how envelope formation is regulated by tau phosphorylation in the presence of other cellular factors, we generated cell extracts^22,23^ by either overexpressing GFP–tau in HEK293T cells (denoted as HEK lysate) or GFP–tau together with Cdk5 and its activator p25 (denoted as Cdk5 lysate) (Extended Data Fig. 4a and Methods). Here and in further cellular experiments, we used p25 (a truncated version of p35) as it has a longer half-life in cells and is not membrane bound^24,25^. To confirm the increased phosphorylation degree, we determined the phosphorylation levels of tau from HEK and Cdk5 lysates at multiple phosphorylation sites using phosphospecific antibodies (Extended Data Fig. 4b–d). We then added the lysates to taxol-stabilized microtubules and followed the tau–microtubule interaction using TIRF microscopy (Extended Data Fig. 4e). We observed tau envelopes forming in the HEK lysate while no envelopes formed in the Cdk5 lysate (Extended Data Fig. 4e,f). These observations are in line with our in vitro experiments showing that phosphorylation of tau decreases the propensity of tau to form envelopes.

Next, we overexpressed GFP–tau and mScarlet–tubulin in U-2 osteosarcoma (OS) cells and followed the tau signal correlated to the microtubule signal throughout the cell cycle, which is tightly regulated by activation of specific proline-directed CDKs known to phosphorylate tau. In line with previous data^26,27^, we observed a high tau signal on microtubules in interphase cells, where the overall activity of kinases is low, and a significant reduction in the tau signal on microtubules in mitotic cells (Extended Data Fig. 4g–i), where multiple mitotic kinases are active^28^, while tau expression levels remained comparable (Extended Data Fig. 4j). While the complexity of the system does not allow for directly linking these results with increased tau phosphorylation, this observation is in agreement with our in vitro findings and published data of decreased affinity of phosphorylated tau for microtubules^10,11^.

Tau proteins either bind microtubules as independent molecules with high turnover (at low concentrations) or, upon crossing a certain threshold density of tau protein on the microtubules (at high concentrations), tau molecules can bind cooperatively, interacting with each other and constituting a slow-turnover, cohesive envelope^12–14^. To discern these two modes of tau–microtubule interaction, we used tau concentrations in the low-nanomolar range in our in vitro assays, allowing the assembly of tau envelopes only partially covering the microtubule, adjacent to microtubule sections covered by independently binding tau molecules. At micromolar concentrations in vitro, microtubules are covered entirely by envelopes of cooperatively binding tau molecules, which can be demonstrated, for example, by its functional consequence, namely the resulting protection of microtubules against the severing enzyme katanin (Extended Data Fig. 3a)^13^. As physiological tau concentrations are in the micromolar range^29^, we expect tau in living cells to uniformly cover the entire microtubule with a continuous envelope of cooperatively binding tau molecules.

**Extended Data Fig. 5:**
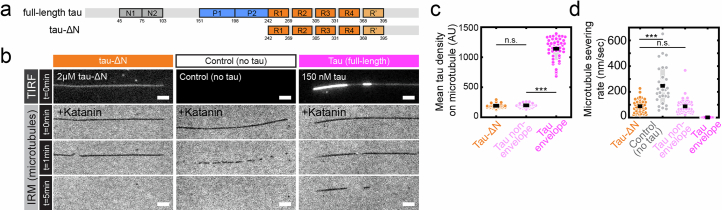
N-terminal truncated tau does not form envelopes a. Schematics of the amino acid sequence of full-length tau (aa 1-441), and tau-∆N (aa 242-441). **b**. Fluorescence micrographs of taxol-stabilized microtubules covered with 2 µM tau-∆N-GFP, no tau (control), or 150 nM full-length tau-GFP (phospho-tau) (top panels). 350 nM Katanin-GFP was added in presence of tau (or control) and microtubule severing was followed using IRM (bottom panels) for 5 min. The inability of tau-∆N to bind cooperatively to microtubules, is substantiated by its lack of forming high density regions on taxol-stabilized microtubules at elevated concentrations and inability to protect microtubules against severing by katanin. Scale bars: 2 μm. **c**. Mean tau density on microtubules before katanin addition, measured for GFP-tau-∆N (orange), and full-length tau in the non-envelope (light magenta) and envelope region (magenta). Data is presented as mean ± s.d. (n = 40, 45, 45 in 5, 6, 6 independent experiments). Two-sided t-test p-values (left-to-right): p = 0.460162, 5.78*10-52. **d**. Microtubule severing (or disassembly) rate (nm/sec) for microtubules covered with tau-∆N-GFP (orange), no tau (control, grey), or full-length tau in the non-envelope (light magenta) or envelope region (magenta). Data is presented as mean ± s.d. (n = 30, 40, 45, 45 microtubules in 3, 5, 6, 6 independent experiments). Two-sided t-test p-values (left-to-right): p = 1.88*10-8, 0.905766. Source data for c and d is available with this manuscript. Stars for statistical tests represent: *** p < 0.001. Source data

**Extended Data Fig. 6:**
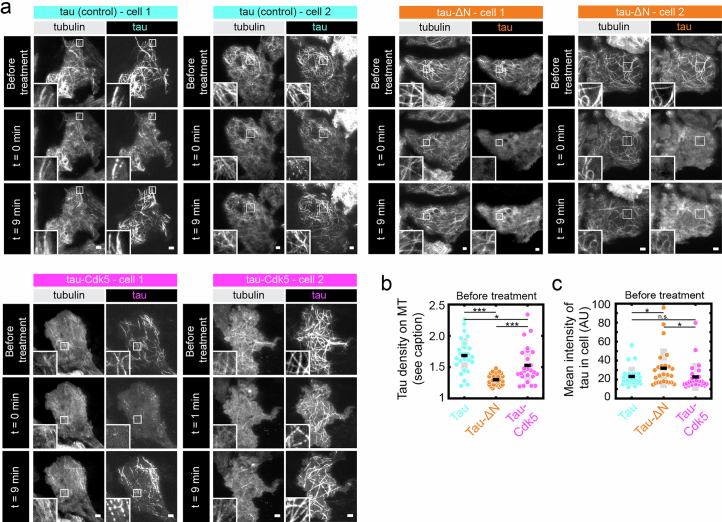
Tau-microtubule interaction is sensitive to the pH of the cell. **a**. Fluorescence micrographs of IMCD-3 cells before elevated-pH treatment (top), at t = 0 min after elevated-pH treatment (middle) and t = 9 min after elevated-pH treatment (bottom). Types of cells subjected to the pH treatment: control GFP-tau cells (tau, cyan), GFP-tau-∆N cells (tau-∆N, orange), and GFP-tau + Cdk5/p25 cells (tau-Cdk5, magenta). Left panels show the mScarlet-tubulin signal, right panels the GFP-tau signal. Scale bars: 10μm. **b**. Density of tau on microtubules before elevated-pH treatment compared to density of tau in the cytoplasm, to give a measure of the binding affinity of tau in the different cells. Data is presented as mean ± s.d. (n = 25 cells for each group in 4 independent experiments). Two-sided t-test p-values (left-to-right): p = 3.26*10-8, 0.0594, 5.89*10-4. **c**. Mean intensity of tau before elevated pH-treatment, as an indication of the expression level, presented as mean ± s.d. (n = 25 cells for each group in 4 independent experiments). Two-sided t-test p-values (left-to-right): p = 0.0658, 0.8901, 0.0774. Source data for **b** and **c** is available with this manuscript. Stars for statistical tests represent: * p < 0.05, *** p < 0.001. Source data

To test this and to assess the regulatory role of tau phosphorylation in cells, we overexpressed GFP–tau together with Cdk5/p25 in IMCD-3 cells, denoted as tau-Cdk5. In a control experiment, we overexpressed GFP–tau in the absence of Cdk5/p25, denoted as tau (control). Additionally, we overexpressed N-terminally truncated GFP–tau (Extended Data Fig. 5a and Methods), denoted as tau-∆N, which is a construct of tau that is not able to form envelopes^12,13^ (Extended Data Fig. 5b–d). Imaging tau localization in cells overexpressing tau, tau-∆N and tau-Cdk5, we found that tau covered microtubules uniformly along their entire lengths (Extended Data Fig. 6a, ‘before treatment’). However, in tau-∆N and tau-Cdk5 cells, the tau signal on the microtubule was weaker (Extended Data Fig. 6b), while the expression levels were comparable (Extended Data Fig. 6c), suggesting that, in tau-∆N and tau-Cdk5 cells, tau has a lower affinity for the microtubule surface or is bound at lower density because of reduced cooperativity between the tau molecules.

**Fig. 3:**
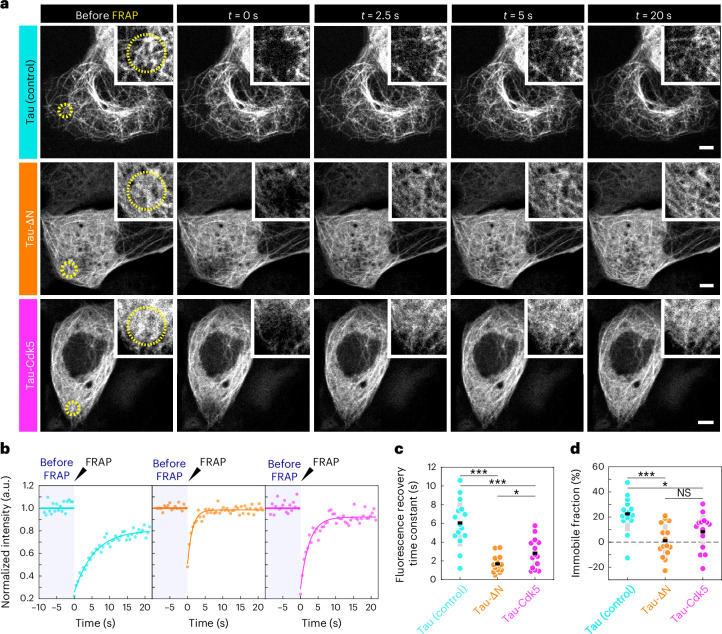
Tau phosphorylation increases turnover of tau in cells. **a**, Fluorescence micrographs of FRAP experiment on IMCD-3 cells expressing GFP–tau (control; cyan), GFP–tau-∆N (tau-∆N, tau 242–441; orange) or GFP–tau with Cdk5/p25 (tau-Cdk5; magenta) at different time points before and after FRAP. Left: the FRAP region is drawn as a yellow dotted circle (before FRAP). Top right: a zoomed-in view containing the FRAP region is shown for each micrograph. Scale bars, 5 µm. **b**, Fluorescence recovery curves after FRAP for control GFP–tau cells (left; cyan), GFP–tau-∆N cells (middle; orange) and GFP–tau-Cdk5 cells (right; magenta). Normalized tau intensity within the FRAP area is plotted over time. The shaded blue area marks the period before FRAP; the black arrowhead indicates the time point at which FRAP occurred. **c**, Time constant of the fluorescence recovery of the tau signal in the different groups, presented as the mean ± s.d. (*n* = 15, 15 and 14 cells in 15, 15 and 14 independent experiments, respectively). Two-sided *t*-test *P* values (left to right): *P* = 6.76 × 10^−7^, 0.00034 and 0.0242. **d**, Immobile fraction measured from the fluorescence recovery curve, presented as the mean ± s.d. (*n* = 15, 15 and 14 cells in 15, 15 and 14 independent experiments, respectively). Two-sided *t*-test *P* values (left to right): *P* = 0.000167, 0.0114 and 0.179. *P < 0.05 and ***P < 0.001; NS, not significant. Source data

To assess the turnover of tau on microtubules in our differently transfected cells, we used fluorescence recovery after photobleaching (FRAP). We photobleached a circular region of the cell and studied the recovery of the tau signal on the microtubules over time (Fig. 3a,b and Supplementary Videos 7–9). We found that the recovery of the tau signal was slowest and that of the immobile fraction was highest in our control cells expressing full-length tau, whereas, in cells expressing tau-∆N, which does not form envelopes, the recovery was fastest and there was no detectable immobile fraction (Fig. 3c,d). Interestingly, in tau-Cdk5 cells the recovery time and immobile fraction of the tau signal fell between that of the control cells and the tau-∆N cells. As cooperatively bound tau shows low turnover^12,13^ and the recovery time and immobile fraction can be used as a readout for protein turnover, these data further support our in vitro findings and suggest that phosphorylated tau has a weaker tau–microtubule interaction, possibly in combination with reduced cooperativity in living cells.

### Tau phosphorylation affects envelope formation in cells

**Fig. 4:**
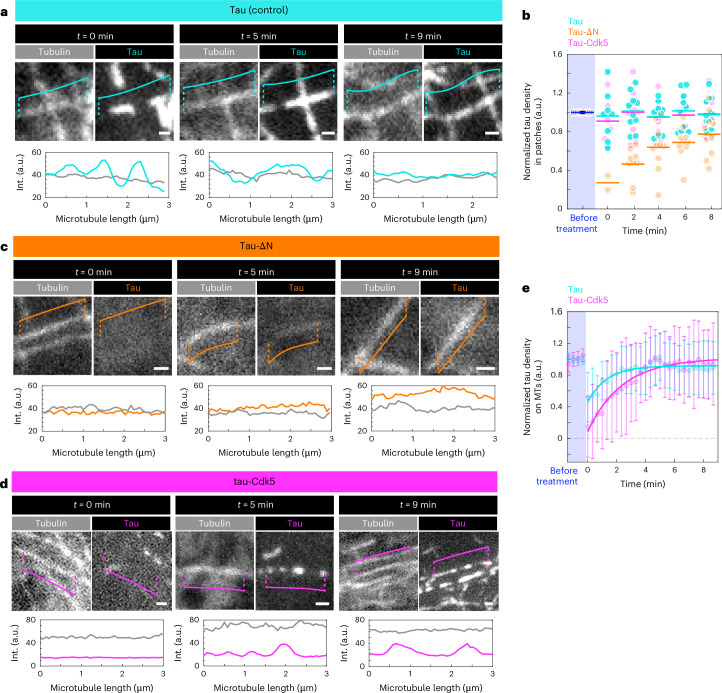
Tau phosphorylation affects envelope formation in cells. **a**, Fluorescence micrographs of IMCD-3 cells expressing mScarlet–tubulin and GFP–tau in control cells (tau; cyan) at *t* = 0 min (left), *t* = 5 min (middle) and *t* = 9 min (right) after elevated-pH treatment. Corresponding linescans are shown below the micrographs. Different microtubules were selected at different time points because of dynamic microtubule behavior. Scale bars, 1 µm. **b**, Tau density in patches of GFP–tau control cells (tau; cyan) and GFP–tau-Cdk5/p25 cells (tau-Cdk5; magenta), normalized to the tau density along the microtubules before treatment (dark blue). In GFP–tau-∆N cells (tau 242–441; orange), normalized tau density is measured along the microtubule lattice (no patches are visible in tau-∆N cells). Data are presented as the mean (bar) and all individual data points (*n* = 10 microtubules). **c**, Fluorescence micrographs of mScarlet–tubulin and GFP–tau signal in tau-∆N cells (orange) at *t* = 0 min (left), *t* = 5 min (middle) and *t* = 9 min (right) after elevated-pH treatment. Corresponding linescans are shown below the micrographs. Different microtubules were selected at different time points because of dynamic microtubule behavior. Scale bars, 1 µm. **d**. Fluorescence micrographs of mScarlet–tubulin and GFP–tau signal in tau-Cdk5 (magenta) at *t* = 0 min (left), *t* = 5 min (middle) and *t* = 9 min (right) after elevated-pH treatment. Corresponding linescans are shown below the micrographs. Different microtubules were selected at different time points because of dynamic microtubule behavior. Scale bars, 1 µm. **e**, Time trace of tau density on microtubules after elevated-pH treatment normalized to the tau density on microtubules before the treatment in GFP–tau cells (tau; cyan) and GFP–tau-Cdk5/p25 cells (tau-Cdk5; magenta). Data are presented as the mean ± s.d. at each time point (*n* = 25 cells) and fitted with an exponential curve (Methods). Exponential time constant was 1.3 min for tau cells and 2.5 min for tau-Cdk5 cells (Methods). *P < 0.05 and ***P < 0.001. Int., intensity. Source data

**Extended Data Fig. 7:**
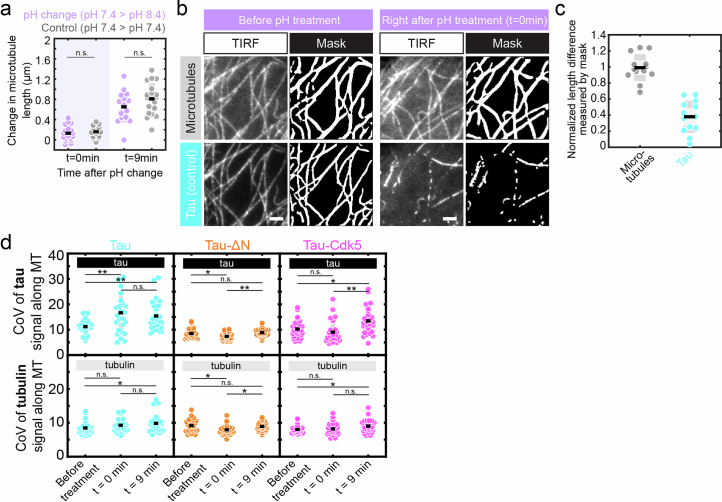
Microtubules remain stable during pH treatment. **a**. Change in length of glycerol-stabilized microtubules immediately after buffer change (left) and 9 min later (right). Buffer was switched from pH 7.4 to pH 8.4 (purple), or pH 7.4 to pH 7.4 (control, grey). Data is presented as mean + s.d. (n = 3, 3 independent experiments with N = 19, 16 microtubules). Two-sided t-test p-values (left to right): p = 0.9417, 0.8982. **b**. Fluorescence micrographs of IMCD-3 cells before elevated pH-treatment (left), and right after elevated pH-treatment (t = 0 min, right). The generated mask from the microtubule signal (mScarlet-tubulin) and corresponding tau signal (GFP-tau, control) is shown next to the image (Methods). Scalebars: 2μm. **c**. Length of microtubule signal (grey) or tau signal (cyan) right after elevated pH-treatment normalized to the length before pH treatment, presented as mean ± s.d. (n = 13 areas in 7 cells). Two-sided t-test, p = 1.17238*10-8. **d**. Coefficient of variation (CoV) of GFP-tau signal (top) or mScarlet-tubulin signal (bottom) measured along the microtubule lattice in GFP-tau (cyan), GFP-tau-∆N (orange), and GFP-tau-Cdk5 (magenta) cells. CoV was measured at 3 timepoints: before elevated-pH treatment (left), at t = 0 min after treatment (middle), and at t = 9 min after treatment (right). Data is presented as mean ± s.d. (CoV of tau, n = 25, 25, 25 cells; CoV of tubulin, n = 25, 25, 25 cells). Two-sided t-test p-values (CoV tau left-to-right): p = 0.0032423, 0.002724, 0.541674, 0.035050, 0.446571, 0.001353, 0.279565, 0.017556, 0.002562; (CoV tubulin left-to-right): p = 0.147458, 0.044724, 0.395936, 0.012266, 0.539248, 0.017755, 0.733709, 0.040452, 0.125798. Source data for **a**, **c** and **d** is available with this manuscript. Stars for statistical tests represent: * p < 0.05, ** p < 0.01. Source data

To assess tau envelope cohesion in living cells, we brought the tau–microtubule interaction out of equilibrium to observe the disassembly or reassembly of tau envelopes. Tau–microtubule binding is sensitive to pH of the environment in vitro and in cells^30,31^. As the stability of GDP-lattice microtubules in vitro was not affected by changing the pH (Extended Data Fig. 7a and Methods), we used a pH change (from pH 7.4 to pH 8.4; Methods) in vivo to shift the tau–microtubule interaction out of equilibrium and temporarily cause unbinding of tau from microtubules. In these conditions, any tau molecules noncooperatively bound to the microtubule would be readily released from microtubules (because of their high turnover), whereas cooperatively bound tau molecules forming envelopes or tau with much higher affinity to the microtubules would be more resilient to unbinding. Indeed, in our positive control cells expressing full-length GFP–tau (denoted as ‘tau’), we observed gaps forming in the tau signal directly after the pH change (Fig. 4a, *t* = 0 min after pH change; whole-cell images in Extended Data Figs. 6a and 7b,c), while the microtubule length was unaffected (Extended Data Fig. 7b,c). This effect was manifested as an increase in the coefficient of variation, a measure of the variance in the tau signal along the microtubules (Extended Data Fig. 7d). These gaps in the tau signal left clearly separated tau patches on the microtubules, strongly suggesting that the unbinding of tau revealed the presence of cooperatively bound tau constituting tau envelopes (Fig. 4a, *t* = 0 min after pH change) and confirming previous observations where taxol treatment led to dissociation of tau from microtubules in cells in a similar patch-like pattern^14^.

The advantage of the elevated-pH treatment compared to the taxol treatment is that tau unbinding is faster and reversible, which allows us to analyze the rebinding of tau to microtubules. Presumably because of a recovery of the intracellular pH after the treatment, the gaps in the tau signal along the microtubules started to close and the tau patches regrew (Fig. 4a, *t* = 5 and 9 min after pH change), while the density of the GFP–tau signal (GFP intensity per unit length) within the patches remained constant (Fig. 4b). This process is analogous to the growth of envelopes in vitro, when excess tau is available^12–14^, suggesting that these patches represent tau envelopes (which grow and shrink only at the boundaries^12,13^). Within 9 min after the elevated-pH treatment, microtubules regained full tau coverage (Fig. 4a; whole-cell images in Extended Data Figs. 6a and 7d; whole-cell treatment in Supplementary Video 10), suggesting that the whole lengths of microtubules were now covered by tau envelopes.

When we performed the elevated-pH treatment on tau-∆N cells (incapable of envelope formation), we did not detect any tau patches on the microtubules after the elevated-pH treatment (Fig. 4c, *t* = 0 min). Instead, we observed that the tau signal was removed uniformly and fully along the entire lengths of the microtubules (Fig. 4c; whole-cell images in Extended Data Figs. 6a and 7d; whole-cell treatment in Supplementary Video 11), which is expected for tau molecules that were bound to the microtubules individually, noncooperatively. Subsequently, after elevated-pH treatment, we observed that the tau-∆N signal returned as a uniform layer along the microtubule lattice and, unlike full-length tau molecules, the density of tau-∆N signal on the microtubule increased uniformly during recovery (Fig. 4b,c; whole-cell images in Extended Data Figs. 6a and 7d; whole-cell treatment in Supplementary Video 11). This further suggests that ∆N-tau, in these cells, does not form envelopes and instead binds to microtubules noncooperatively (that is, with high turnover and high diffusivity).

In the tau-Cdk5 cells, we observed that the reappearance and recovery of the signal after elevated-pH treatment occurred in a patch-like manner, closely resembling the reappearance of the tau signal in our positive control cells (Fig. 4d; whole-cell images in Extended Data Figs. 6a and 7d; whole-cell treatment in Supplementary Video 12). Moreover, the density of the tau signal within the patches remained constant throughout the treatment (Fig. 4b), indicating that tau was bound cooperatively to the microtubules in tau-Cdk5 cells. Interestingly, the reappearance of the tau signal on microtubules was slower in tau-Cdk5 cells compared to the control cells, indicating that tau phosphorylation leads to slower envelope recovery, which could be because of a reduced tau–microtubule interaction, possibly in combination with decreased tau cooperativity (Fig. 4e). Combined, these data suggest that, in control and tau-Cdk5 cells, tau is bound cooperatively, forming cohesive tau envelopes, while N-terminally truncated tau in tau-∆N cells binds noncooperatively. The fact that, after the elevated-pH treatment, the tau signal disappeared almost completely and reappeared more slowly in the tau-Cdk5 cells compared to control tau cells is in agreement with our in vitro data showing that tau phosphorylation does not prevent tau envelope formation but makes tau less prone to form envelopes and makes the resulting envelopes less stable. Combined, our data suggest that tau phosphorylation decreases the cohesiveness of tau envelopes, thereby negatively affecting the stability of the tau envelopes in living cells.

### Tau phosphorylation affects envelope functionality in vitro

**Fig. 5:**
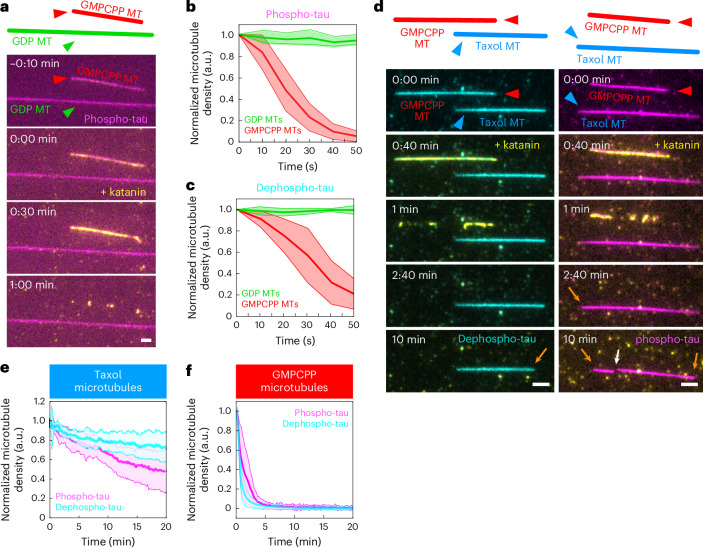
Tau phosphorylation affects envelope functionality in vitro. **a**, Schematics and multichannel fluorescence micrographs of 120 nM phospho-tau on GMPCPP (red) and GMPCPP-capped GDP (green) microtubules. MT, microtubule. Here, 100 nM katanin–GFP was added at *t* = 0 min in the presence of tau, leading to severing of GMPCPP microtubules while GDP microtubules remained protected. Experiments were performed six times for both dephospho-tau and phospho-tau, yielding similar results. Scale bar, 2 μm. **b**, Normalized microtubule density over time for GMPCPP (red) and GDP (green) microtubules covered by phospho-tau envelopes after the addition of katanin at *t* = 0 s. Data of GMPCPP and GDP microtubules are presented as the mean (center line) ± s.d. (shaded area) (*n* = 25 and 40 microtubules, respectively, in six independent experiments). **c**, Normalized microtubule density over time as in **b**, but with dephospho-tau envelopes. Data of GMPCPP and GDP microtubules are presented as the mean (center line) ± s.d. (shaded area) (*n* = 9 and 29 microtubules, respectively, in six independent experiments). **d**, Fluorescence micrographs of 30 nM dephospho-tau (left; cyan) or 120 nM phospho-tau (right; magenta) on GMPCPP (red) and taxol-stabilized (blue) microtubules. Here, 500 nM katanin–GFP was added at *t* = 40 s, leading to GMPCPP microtubules severing while taxol-stabilized microtubules remained protected. Katanin severing eventually led to disassembly of envelope-covered portions of taxol-stabilized microtubules mainly from their boundaries (orange arrows), with occasional katanin severing within a tau envelope (white arrow). Experiments were performed ten (dephospho-tau) and eight (phospho-tau) times, yielding similar results. Scale bars, 2 μm. **e**, Normalized microtubule density over time for taxol-stabilized microtubules covered with either 120 nM phospho-tau (magenta) or 30 nM dephospho-tau (cyan). Data for phospho-tau and dephospho-tau are presented as the mean (center line) ± s.d. (shaded area) (*n* = 12 and 19 microtubules, respectively, in eight and ten independent experiments). **f**, Normalized microtubule density over time for GMPCPP microtubules covered with either 120 nM phospho-tau (magenta) or 30 nM dephospho-tau (cyan). Data for phospho-tau and dephospho-tau are presented as the mean (center line) ± s.d. (shaded area) (*n* = 16 and 28 microtubules, respectively, in eight and ten independent experiments). Source data

**Extended Data Fig. 8:**
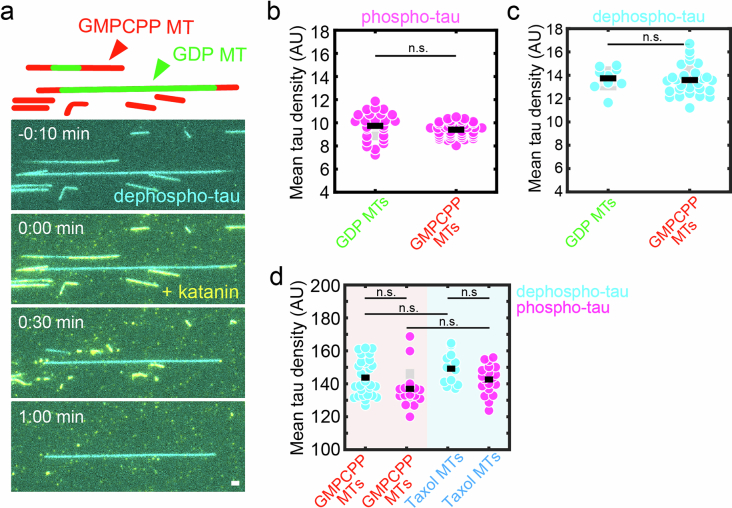
Tau phosphorylation affects envelope functionality in vitro. **a**. Multichannel fluorescence micrographs (schematics above) of 30 nM dephospho-tau (cyan) on GMPCPP (red arrowhead) and GMPCPP-capped GDP (green arrowhead) microtubules. 100 nM katanin-GFP is added at t = 0 min after which rapid disintegration of the GMPCPP microtubule can be observed while the GDP microtubule is protected against katanin severing. Scale bar: 2 μm. **b**. Comparable mean phospho-tau density (magenta) on GMPCPP-capped GDP microtubules (green) and GMPCPP microtubules (red) incubated with 120 nM phospho-tau. Data is presented as mean ± s.d. (n = 25, 40 microtubules in 6, 6 independent experiments). Two-sided t-test, p = 0.153812. **c**. Comparable mean dephospho-tau density (cyan) on GMPCPP-capped GDP microtubules (green) and GMPCPP microtubules (red) incubated with 30 nM dephospho-tau. Data is presented as mean ± s.d. (n = 9, 29 microtubules in 5, 5 independent experiments). Two-sided t-test, p = 0.759219. d. Comparable mean tau density on GMPCPP microtubules (red) and taxol-stabilized microtubules (blue) incubated with 120 nM of phospho-tau (magenta) or 30 nM dephospho-tau (cyan). Data is presented as mean ± s.d. (n = 28, 16, 12, 19 microtubules in 10, 8, 10, 8 independent experiments). Two-sided t-test p-values (left to right) p = 0.062914, 0.144124, 0.12011, 0.064036. Source data for **b**, **c** and **d** is available with this manuscript. Source data

Finding that tau envelopes could be formed by tau in both phosphorylated and nonphosphorylated states, albeit at different efficiencies, and that phosphorylation leads to decreased tau cooperativity and compromised envelope integrity, we asked whether tau phosphorylation additionally affects the functionality of the envelopes. Tau envelopes regulate the accessibility of the microtubule surface for other microtubule-associated proteins and can protect microtubules against the action of microtubule-severing enzymes such as katanin^12,13^. To confirm that specifically tau envelopes can protect microtubules against katanin severing, we used GMPCPP microtubules that prevent tau from forming envelopes^12,14^. We surface-immobilized both GMPCPP microtubules and GMPCPP-capped GDP microtubules on a coverslip surface and added 120 nM phospho-tau or 30 nM dephospho-tau to reach a similar density of tau on both microtubule types (Fig. 5a, Extended Data Fig. 8a–c and Supplementary Videos 13 and 14). We then added katanin in presence of tau to the channel and observed that, while GMPCPP microtubules (no tau envelope formed) quickly disintegrated, GDP microtubules (covered with tau envelope) remained unsevered (Fig. 5b,c and Supplementary Videos 13 and 14), confirming that tau envelopes specifically protect microtubules against katanin severing.

**Extended Data Fig. 9:**
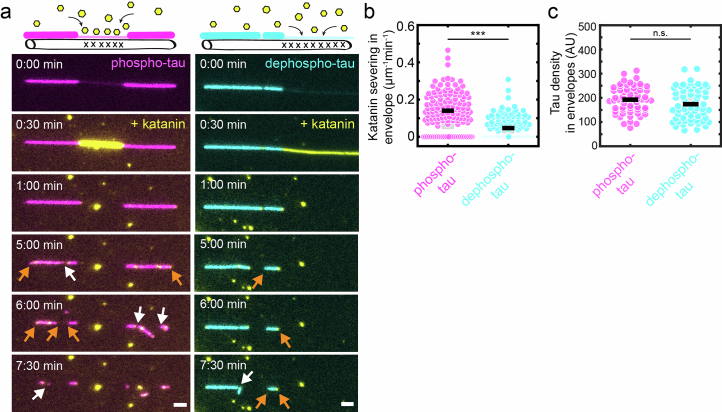
Tau envelopes protect microtubules against katanin severing. **a**. Multichannel fluorescence micrographs (schematics above) of 30 nM phospho-tau (magenta, left) or 5 nM dephospho-tau (cyan, right) showing rapid katanin-GFP-mediated severing of microtubule regions not covered by a tau envelope (panel 2, tau in magenta/cyan, katanin in yellow), followed by much slower disassembly of envelope-covered microtubule regions (panels 3-6). Katanin severing from the boundaries of tau envelopes is indicated by orange arrows; occasional severing within a tau envelope (severing event) is indicated by white arrows. Scale bars: 2 μm. **b**. Katanin severing events within tau envelope boundaries in phospho-tau envelopes (magenta) and dephospho-tau envelopes (cyan), presented as mean ± s.d. (n = 127, 132 envelopes in 4, 4 experiments). Two-sided t-test, p = 1.85*10-17. **c**. Comparable tau density within envelopes constituted of 30 nM phospho-tau (magenta) or 5 nM dephospho-tau (cyan), presented as mean ± s.d. (n = 60, 60 envelopes in 3, 3 independent experiments). Two-sided t-test, p = 0.097192. Source data for **b** and **c** is available with this manuscript. Stars for statistical tests represent: *** p < 0.001. Source data

As GDP microtubules quickly disassembled because of microtubule instability, once their GMPCPP caps were disintegrated by katanin, we were unable to study the effect of tau phosphorylation on microtubule protection over longer timescales. To achieve this, we repeated the experiment by surface-immobilizing GMPCPP microtubules (no envelope formation) and taxol-stabilized microtubules (allowing envelope formation) and added 30 nM dephospho-tau or 120 nM phospho-tau to reach similar densities of tau on both microtubule types (Extended Data Fig. 8d). We then added katanin in presence of tau to the channel and observed that, while GMPCPP microtubules quickly disintegrated, taxol-stabilized microtubules remained unsevered (Fig. 5d and Supplementary Videos 15 and 16), confirming our previous findings that specifically tau envelopes can protect microtubules against katanin severing. When comparing the effect of phosphorylation on the protective functionality of tau, we found that the ability of phospho-tau to protect taxol-stabilized microtubules was reduced compared to dephospho-tau (Fig. 5e), while the inability to protect GMPCPP microtubules was comparable between phospho-tau and dephospho-tau (Fig. 5f), showing a decrease in protective functionality of phosphorylated tau envelopes. Strikingly, when following the disassembly of tau envelopes on taxol-stabilized microtubules, we observed an increase in the number of katanin severing events within the envelope regions prepared with phospho-tau (Extended Data Fig. 9a,b) compared to tau envelopes prepared with dephospho-tau at similar tau densities (Extended Data Fig. 9c), which may be explained by the reduced tau envelope integrity because of decreased affinity and reduced cooperativity of phosphorylated tau, as shown above. Combined, these data demonstrate that an increase in phosphorylation results in a reduction in the protective functionality of tau envelopes.

### Tau phosphorylation affects envelope functionality in vivo

**Fig. 6:**
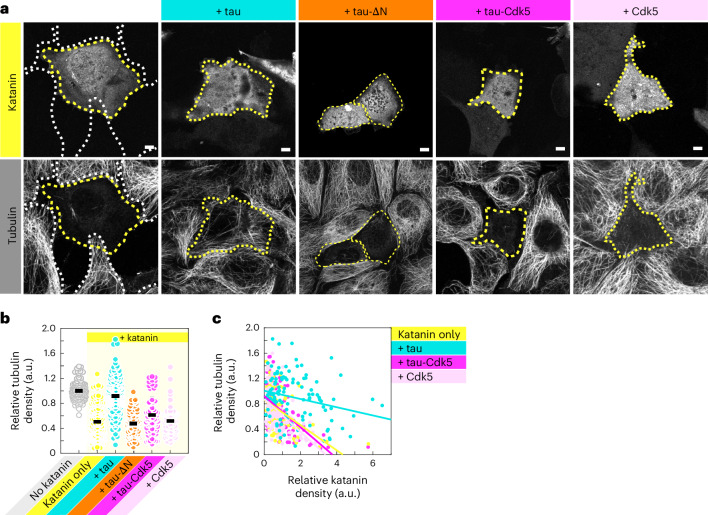
Tau phosphorylation affects envelope functionality in vivo. **a**, Fluorescence micrographs of IMCD-3 cells expressing katanin–GFP (top), fixed 12 h after transfection and stained for tubulin (bottom). In combination with katanin, cells express full-length mCherry–tau (+tau; cyan), mCherry–tau-∆N (+tau-∆N; orange), mCherry–tau and Cdk5/p25 (+tau-Cdk5; magenta) or Cdk5/p25 in the absence of tau (+Cdk5; light pink). Cells expressing katanin are marked with yellow dotted lines and cells not expressing katanin (nontransfected cells) are marked with white dotted lines. Scale bars, 5 µm. Experiments were performed three times for each cell type, yielding similar results. **b**, Relative tubulin density 12 h after transfection, presented as the mean ± s.d. (*n* = 132, 131 and 132 cells in six independent experiments or 60, 60 and 72 cells in three independent experiments). **c**, Correlation of the relative tubulin density (*y* axis) compared to the relative katanin density (*x* axis). Correlation coefficients were −0.49 (katanin only; yellow), −0.25 (+tau; cyan), −0.57 (+tau-Cdk5; magenta) and −0.54 (+Cdk5; light pink). Source data

**Extended Data Fig. 10:**
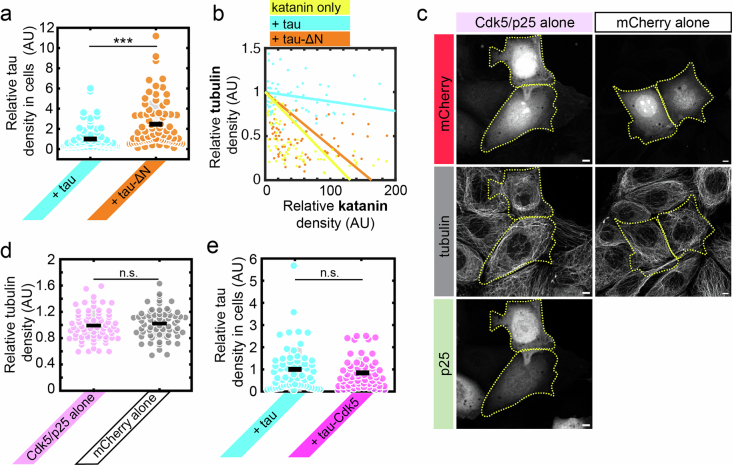
Tau phosphorylation affects envelope functionality in vivo. **a**. Relative tau density in cells expressing Katanin-GFP in combination with mCherry-tau ( + tau, cyan) or mCherry-tau-∆N ( + tau-∆N, orange). Tau densities were set to 1 for tau cells and compared to the densities of tau-∆N cells, presented as mean ± s.d. (n = 75, 75 cells in 3, 3 independent experiments). Two-sided t-test, p = 3.3153*10-6. **b**. Correlation of the relative tubulin density (y-axis) compared to the relative katanin density (x-axis). Correlation coefficients of ‘katanin only‘ is -0.008 (yellow), of ‘+ tau‘ is -0.001 (cyan), and of ‘+ tau-∆N‘ is -0.006 (orange). **c**. Fluorescence micrographs of IMCD-3 cells co-transfected with mCherry in combination with Cdk5/p25 to visualize transfected cells (‘Cdk5/p25 alone’, left panels) or with mCherry in combination with an empty vector (control, ‘mCherry alone’, right panels). Cells were fixed and stained 12 hours after transfection with anti-tubulin (grey, middle panels) and anti-p25 antibodies (green, bottom panel). Cells expressing mCherry are marked with a yellow dotted line. Scale bars: 5 µm. **d**. Relative tubulin density in IMCD-3 cells transfected with mCherry in combination with Cdk5/p25 (‘Cdk5/p25 alone’, light pink) or mCherry and empty vector (‘mCherry alone’, grey). Data is presented as mean ± s.d. (n = 63, 67 cells in 2, 2 independent experiments). Two-sided t-test p = 0.46689. e. Relative tau density in cells expressing mCherry-tau (tau, cyan) or mCherry-tau + Cdk5/p25 (tau-Cdk5, magenta). Tau densities were set to 1 for tau cells and compared to the densities of tau-Cdk5 cells (mean ± s.d., n = 75, 75 cells in 3, 3 independent experiments). Two-sided t-test, p = 0.319199. Source data for **a**, **b**, **d** and **e** is available with this manuscript. Stars for statistical tests represent: *** p < 0.001. Source data

We next asked whether tau envelopes protect microtubules in cells. To test this, we prepared IMCD-3 cells overexpressing katanin (denoted as ‘katanin only’) or katanin in combination with either full-length tau (denoted as ‘+tau’) or the N-terminally truncated construct of tau, tau-∆N (denoted as ‘+tau-∆N’). Cells were fixed 12 h after transfection and stained for tubulin to visualize the presence of microtubules (Methods). In cells expressing katanin in the absence of tau (Fig. 6a, transfected cells, yellow marker), a clear reduction in tubulin signal was observed in comparison to cells not expressing katanin (Fig. 6a,b, nontransfected cells, white marker). Consistent with previously published data^5^, in cells expressing katanin in combination with tau, no reduction in tubulin signal was measured (Fig. 6a,b), indicating that full-length tau protects microtubules against the severing activity of katanin, which is in agreement with our in vitro data. Strikingly, in cells expressing katanin in combination with the noncooperatively binding tau construct tau-∆N, the tubulin signal was reduced similarly to cells expressing katanin alone (Fig. 6a,b), while expression levels of tau-∆N exceeded the expression levels of full-length tau (Extended Data Fig. 10a). Comparing the relative density of tubulin to the relative density of katanin 12 h after transfection, we found that the tubulin density was inversely correlated with the katanin density in all cell groups (Fig. 6c). This correlation was weaker in the presence of full-length tau and stronger in the presence of N-terminally truncated tau or in the absence of tau (Extended Data Fig. 10b). Combined, these data support the notion that tau envelopes protect microtubules against the severing activity of katanin in cells.

We next asked whether tau phosphorylation affects the functionality of envelopes in living cells. To test this, we prepared IMCD-3 cells overexpressing katanin in combination with tau and Cdk5/p25 (denoted as ‘+tau-Cdk5’). As overexpression of Cdk5/p25 affects the phosphorylation of many targets in cells, we prepared control IMCD-3 cells overexpressing katanin in combination with Cdk5/p25 (denoted as ‘+Cdk5’) and IMCD-3 cells overexpressing Cdk5/p25 in the absence of katanin (denoted as ‘Cdk5/p25 alone’; Extended Data Fig. 10c) and its control (denoted as ‘mCherry alone’; Extended Data Fig. 10c). Studying the tubulin density 12 h after transfection, we found that cells overexpressing Cdk5/p25 in absence of katanin and tau did not affect the tubulin signal (Extended Data Fig. 10c,d). Likewise, overexpression of katanin in presence of Cdk5/p25 (‘+Cdk5’) similarly reduced the tubulin signal compared to overexpression of katanin alone (Fig. 6a,b), concluding that Cdk5/p25 overexpression does not affect microtubule stability. Interestingly, overexpressing katanin in combination with tau and Cdk5/p25 (‘+tau-Cdk5’) showed a clear reduction in the tubulin signal compared to cells overexpressing katanin in combination with tau in absence of Cdk5/p25 (‘+tau’), while the tau expression levels were comparable (Fig. 6a,b and Extended Data Fig. 10e). Comparing the relative density of tubulin to the relative density of katanin 12 h after transfection, we found that tubulin density was more strongly inversely correlated with katanin density in the presence of tau-Cdk5 compared to tau in the absence of Cdk5 (Fig. 6c). These results suggest that phosphorylation of tau by Cdk5 impedes the protective functionality of the tau envelopes in cells.

## Discussion

Tau envelopes are cohesive patches of cooperatively binding tau molecules characterized by limited lateral translocation and low turnover of the constituting tau molecules^12,13^. These envelopes are selectively permeable for some proteins, while protecting the microtubules from others, for example, the severing enzyme katanin^13^. This mode of tau–microtubule interaction is contrasted by noncooperative tau–microtubule binding, observed in vitro at low tau concentrations^12,13^, when using the N-terminally truncated tau^13^ or on GMPCPP microtubules^12,14^. This noncooperative binding mode of tau is characterized by rapid diffusion and high turnover of the individual tau molecules, which do not shield binding of other proteins and, thus, for example, leave the microtubule vulnerable to severing enzymes^13^. Here, we showed that phosphorylation of tau impedes the envelope formation and negatively impacts envelope integrity and protective functionality. Moreover, the presence of phosphorylated tau leads to disassembly of preexisting tau envelopes. In neurons, this might amplify the reported deleterious effect of tau phosphorylation. Release of tau from envelopes may locally increase cytosolic tau concentration and promote the formation of phosphorylated tau aggregates with additional toxic effects on neuronal transport and function. Additionally, because of the reduced protection of phosphorylated tau envelopes, microtubule destabilization might occur even before tau envelope disassembly.

Previous results showed that tau phosphorylation reduces tau association with microtubules^10,11,18–21^. The affinity of tau for microtubules and the cooperativity of the interaction are closely interconnected, making it challenging to discern one effect from the other, especially in vivo. In our in vitro assays, we could separate the two effects, demonstrating that phosphorylation reduces not only the tau–microtubule affinity but also the cooperativity of the tau–microtubule interaction (Fig. 2c,d). Interestingly, a recent study showed that a different tau phosphorylation pattern decreased the tau–microtubule affinity while increasing the cooperativity of the interaction^32^, suggesting that different tau phosphorylation patterns can differentially regulate the tau–microtubule interaction.

We observed that tau phosphorylation reduced the microtubule-protective function of tau envelopes. This effect could be because of higher turnover of phosphorylated tau (decreased tau–microtubule interactions) or compromised tau cooperativity (decreased tau–tau interactions), which might result in transient defects in the envelope. The latter hypothesis is especially plausible when considering the position of phosphorylation in our phospho-tau samples; while little to no phosphorylation was detected within the binding repeats, which directly interact with the microtubule, most phosphorylation was detected in the projection domains of tau (particularly P1, P2 and the C terminus), which are the regions thought of as establishing the tau–tau interaction^12,13^. Moreover, phosphorylation of Cdk5 sites Ser202, Thr205, Thr231 and Ser235 was previously shown to have a negligible effect on binding of tau to microtubules, suggesting a broader effect of tau phosphorylation only on the microtubule affinity^33^.

Tau is phosphorylated by numerous kinases in healthy neurons; however, deregulation of Cdk5 seems critical in the neuropathological process leading to neurodegeneration. We now demonstrate that upregulation of Cdk5 has a deleterious effect on the formation and maintenance of protective tau envelopes and that microtubules covered by tau envelopes formed by phosphorylated tau are more prone to disintegration, for example, by microtubule-severing enzymes, such as katanin. As we are working with an exogenous system using overexpression of tau and Cdk5, these conditions may differ from neurons. However, using a combination of our in vitro and in vivo approaches, we were able to link Cdk5 upregulation with tau phosphorylation and tau envelope disintegration. Therefore, our work may provide a mechanism of microtubule regulation in cells and in the pathogenesis of neurodegenerative disorders, such as Alzheimer disease. Our results suggest that microtubule destabilization could be the result of the impaired protective functionality of tau envelopes upon phosphorylation of tau.

## Methods

### Protein constructs and purification

#### Insect-cell-expressed tau

For in vitro experiments, GFP-labeled or mCherry-labeled tau (h441-tau; NM_005910.6, 151–1476) or GFP-tagged tau-∆N (h242–441 tau) was expressed in insect cells and purified using the baculovirus expression system (DefBac DNA). Sf9 cells were infected with 8 ml of P2 baculovirus stock (1:100 ratio of P2 virus to cell culture), incubated at 27 °C with moderate shaking and harvested 72–78 h after infection. Cells were harvested by centrifugation at 300*g* for 10 min and resuspended in PBS before snap-freezing the cells or purification in lysis buffer (50 mM HEPES pH 7.4, 2 mM MgCl_2_, 1 mM EGTA, 150 mM KCl and 20 mM imidazole, with 1 mM DTT, Benzonase (1.25 µl of 25 U per µl; 70664, Novagen) and 1× protease inhibitor cocktail (34044100, Roche Diagnostics)). Cells were lysed by spinning at 35,000–70,000*g* for 1 h at 4 °C and collecting the supernatant. The lysate was incubated with Ni-NTA agarose resin (XF340049, Thermo Scientific) HiTrap for 2 h at 4 °C by slowly rotating. After incubation, beads were washed three times with 20 ml of wash buffer (50 mM HEPES, 2 mM MgCl_2_, 1 mM EGTA, 150 mM KCl (or 700 mM KCl in wash step 2), 1 mM DTT and 20 mM imidazole). Then, 6×His tag was removed by incubating the beads with PreScission protease (homemade 3C human rhinovirus protease, 1:100, 1 µg enzyme per 100 µg of protein, overnight at 4 °C while rotating). The next day, the cleaved protein was collected and concentrated by spinning the sample at 3,500 rpm at 4 °C using a 50-kDa (for full-length tau) or 10-kDa (for tau-∆N) centrifugal filter tube (Amicon Ultra-15, Merck). The protein was purified by size-exclusion chromatography using a Superdex 200 10/300 GL column (GE28-9909-44, Sigma) with an NGC chromatography system (Bio-Rad), equipped with ChromLab software (Bio-Rad), in 50 mM HEPES pH 7.4, 2 mM MgCl_2_, 1 mM EGTA, 150 mM KCl, 1 mM DTT, 0.1 mM ATP and 1 mM EDTA. Collected peak fractions were concentrated using a 50-kDa (for full-length tau) or 10-kDa (for tau-∆N) centrifugal filter tube (Amicon Ultra-15, Merck). Protein concentration was measured with a NanoDrop ND-1000 spectrophotometer (Thermo Scientific) at 280-nm absorbance. Proteins were flash-frozen in liquid nitrogen and stored at −80 °C. All steps in the purification were performed at 4 °C.

#### Bacterial cell expressed tau

Fluorescently tagged tau used for in vitro experiments (h441-tau subcloned into the expression vector based on pET11Kan-N-HIS6-3C-mNeonGreen or pET11Kan-N-HIS6-3C-mRuby3) was expressed in *Escherichia* *coli* BL21(DE3)-RIPL strain. The cells were grown at 30 °C until an optical density at 600 nm of 0.5–0.6; the protein expression was then induced by 0.1 mM IPTG and the cells were grown overnight at 16 °C. Bacterial cells (3–4 g) were lysed in 45 ml of lysis buffer (50 mM Tris pH 8.0, 300 mM NaCl, 2 mM β-mercaptoethanol, 20 mM imidazole, 0.5 µL Benzonase and 1× protease inhibitor cocktail), sonicated (5 min; 2 s on, 4 s off) and centrifuged (40,000*g*, 30 min, 4 °C). The soluble fraction was then subjected to Strep-Tactin XT purification (washing buffer: 50 mM Tris pH 8.0, 300 mM NaCl and 2 mM β-mercaptoethanol) and eluted using BXT buffer (100 mM Tris pH 8.0, 150 mM NaCl, 1 mM EDTA and 50 mM biotin). The purified protein was concentrated using VivaSpin-10kDa-HY and subjected to size-exclusion chromatography, as described above (buffer: 50 mM Tris pH 8.0, 300 mM NaCl and 1 mM DTT). Protein concentration was measured with a NanoDrop, as described above, at 280-nm absorbance. Proteins were flash-frozen in liquid nitrogen and stored at −80 °C. All purification steps were performed at 4 °C.

#### Katanin expression and purification

Katanin–GFP^34^ (p60–GFP and p80–GFP) was expressed and purified as previously described.

### TIRF microscopy

TIRF microscopy experiments were performed on an inverted microscope (Nikon-Ti E, Nikon TI2 E) with an H-TIRF module or iLas2 equipped with ×60 or ×100 (numerical aperture: 1.49) oil immersion objectives (Apo TIRF or SR Apo TIRF, respectively, Nikon) and complementary metal–oxide–semiconductor (CMOS) Hamamatsu Orca Flash 4.0 LT, scientific CMOS Hamamatsu ORCA 4.0 V2 or PRIME BSI (Hamamatsu Photonics, Teledyne Photometrics) cameras. Microtubules were visualized using interference reflection microscopy (IRM) and fluorescent proteins by switching among microscope filter cubes for EGFP, mCherry and Cy5 channels or using a quad-band set (405, 488, 561 and 640 nm). The microscopes were controlled with Nikon NIS Elements software (version 5.02, 5.20 or 5.42). All experiments were performed at room temperature by several experimentalists over the course of multiple months. No data were excluded from the study.

#### Experimental chamber preparation

For TIRF experiments, chambers were assembled by melting thin strips of parafilm in between two glass coverslips silanized with 0.05% dichlorodimethylsilane (440272, Sigma) or hexamethyldisilazane (379212, Sigma). The chambers were incubated with 20 µg ml^−1^ anti-biotin antibodies (in PBS; B3640, Sigma) or 20 µg ml^−1^ anti-β-tubulin antibodies (in PBS; T7816, Sigma) for 5 min, followed by 1% Pluronic (F127 in PBS; P2443, Sigma) for at least 30 min. Microtubules were diluted in BRB80T (80 mM PIPES pH 6.9, 1 mM EGTA and 1 mM MgCl_2_, supplemented with 10 µM paclitaxel (17191, Sigma)) and then incubated in the chamber and allowed to adhere to the antibodies. Unbound microtubules were washed away with BRB80T and chambers were preincubated with TIRF assay buffer (AB; 50 mM HEPES pH 7.4, 1 mM EGTA, 2 mM MgCl_2_, 75 mM KCl, 10 mM dithiothreitol, 0.02 mg ml^−1^ casein, 10 µM taxol, 1 mM Mg-ATP, 20 mM D-glucose, 0.22 mg ml^−1^ glucose oxidase and 20 µg ml^−1^ catalase) before experiments. Unless stated otherwise, all experiments were conducted in TIRF AB. All experiments were quantified by pooling data from multiple chambers performed on at least two different days. Chambers were never reused for additional experiments.

#### Microtubule assembly

Porcine brains were obtained from a local abattoir and used within ~4 h of death. Porcine brain tubulin was isolated using the high-molarity PIPES procedure^35^. Biotin-labeled tubulin was purchased from Cytoskeleton (T333P) and diluted 1:50 with unlabeled porcine brain tubulin to obtain biotin-labeled tubulin mix for surface-immobilization assays using biotin antibodies.

Taxol-stabilized microtubules (GTP-polymerized, then taxol-stabilized, stored and imaged in presence of taxol) were polymerized from 4 mg ml^−1^ tubulin for 30 min at 37 °C in BRB80 supplemented with 4 mM MgCl_2_, 5% DMSO and 1 mM GTP (NU-1012, Jena Bioscience). The polymerized microtubules were diluted in BRB80T and centrifuged for 30 min at 18,000*g* in a Microfuge 18 centrifuge (Beckman Coulter). After centrifugation the pellet was resuspended and kept in BRB80T at room temperature.

GMPCPP microtubules (GMPCPP-polymerized, then taxol-stabilized, stored and imaged in presence of taxol) were polymerized from 4 mg ml^−1^ tubulin for 2 h at 37 °C in BRB80 supplemented with 1 mM MgCl_2_ and 1 mM GMPCPP (NU-405, Jena Bioscience). The polymerized microtubules were centrifuged for 30 min at 18,000*g* in a Microfuge 18 xentrifuge (Beckman Coulter). After centrifugation, the pellet was resuspended and kept in BRB80T at room temperature.

GDP microtubules (GTP-polymerized, then glycerol-stabilized, stored and imaged in presence of 40% glycerol) were polymerized as described for taxol-stabilized microtubules. After polymerization, the microtubules were gently diluted in BRB80-Gly40 buffer (80 nM PIPES, 2 mM MgCl_2_, 1 mM EGTA pH 6.8 and 40% glycerol) and centrifuged as described above. After centrifugation, the supernatant was discarded and the pellet was resuspended gently in 50 μl of BRB80-Gly40. Microtubules were then kept at room temperature at least 1 h (maximum overnight) before usage.

GMCPP-capped GDP microtubules (GTP-polymerized and then stabilized by GMPCPP caps) were polymerized and centrifuged as described for taxol-stabilized microtubules. After centrifugation, the pellet was gently resuspended in 40 μl of warm (37 °C) BRB80 supplemented with 1.27 mM MgCl_2_ and 1.25 mM GMPCPP and 0.62 μl of 4 mg ml^−1^ 488-labeled tubulin. GMPCPP caps were polymerized for 20 min at 37 °C. After GMPCPP cap polymerization, microtubules were kept at room temperature and used immediately for in vitro experiments.

#### Tau sample preparation

To study the effect of phosphorylation of tau on envelope formation four samples were produced with various degrees of phosphorylation, as described below. Samples were stored on ice after treatment and directly used for TIRF experiments.

##### Dephospho-tau

Tau was expressed in insect cells, treated with alkaline phosphatase (FastAP phosphatase; EF0651, Themo Fisher). Then, 2 μM (0.2 mg ml^−1^) insect-cell-expressed tau was incubated with 2.5 g mol^−1^ alkaline phosphatase (stock: 10 g mol^−1^) and 1× fast phosphatase buffer for 15 min at 37 °C.

##### Phospho-tau

Tau was expressed in insect cells. Then, 2 μM (0.2 mg ml^−1^) insect-cell-expressed tau was incubated in 1× fast phosphatase buffer in the absence of alkaline phosphatase for 15 min at 37 °C.

##### Bact-Cdk5-tau

Tau was expressed in bacterial cells treated with Cdk5/p35 kinase (V3271, Promega). Then, 2 μM (0.2 mg ml^−1^) Bact-tau was incubated in reaction buffer A (K03-09, stock 5×) supplemented with 50 μM DTT, 50 μM ATP and 0.02 μg μl^−1^ Cdk5/p35 kinase (stock: 0.1 μg μl^−1^) for 15 min at 37 °C.

##### Bact-tau

Tau was expressed in bacterial cells. Then, 2 μM (0.2 mg ml^−1^) Bact-tau was incubated in reaction buffer A (K03-09, stock 5×), in the absence of Cdk5/p35 kinase, supplemented with 50 μM DTT and 50 μM ATP for 15 min at 37 °C.

#### TIRF assays

In all TIRF experiments, chambers were prepared as described above and microtubules were observed using IRM. Unless stated otherwise, taxol-stabilized microtubules were used in the in vitro TIRF experiments. Videos were captured with appropriate frame interval and analysis was performed after a certain incubation time as stated in the figure legends or described below.

##### Tau on microtubules

Tau samples were diluted in AB to the final concentration stated in the text. After microtubule were immobilized on the coverslip surface, the diluted tau sample was added to the measurement chamber with at least a fourfold amount of the chamber volume.

##### Cdk5 treatment in channel

First, 15 nM mNeongreen–tau was diluted in AB buffer (supplemented with 0.5 mg ml^−1^ casein) and incubated on surface-immobilized microtubules for 10 min. After incubation, either active Cdk5/p35 (activity: 0.1 μg μl^−1^, diluted tenfold) or deactivated Cdk5/p35 (deactivated by incubating at 95 °C for 10 min, diluted tenfold) was added to the chamber while tau concentration remained unchanged. Tau envelopes were observed for 15 min.

##### Cdk5 treatment with tau removal

mNeonGreen–tau (bacterial expressed) was diluted in AB to a final concentration of 10–20 nM and incubated on surface-immobilized microtubules for 5 min. After incubation, tau was removed from the channel by flushing in 20 μl of AB in the presence of active Cdk5/p35 (activity: 0.1 μg μl^−1^, diluted tenfold) or deactivated Cdk5/p35 (deactivated by incubating at 95 °C for 10 min, diluted tenfold) or in the absence of any kinase (that is, only AB; control). The disassembly of tau envelopes was observed for 5 min (for active Cdk5), 10–45 min (for deactivated Cdk5) or 10 min (for control).

##### Hill coefficient assay

Different tau concentrations were flushed into measurement chambers containing microtubules, after which the chambers were sealed using wax and imaged after 45 min of incubation to establish equilibrium in tau binding.

##### Tau (1 µM) and katanin assay

First, 1 µM phospho-tau–mCherry was diluted in AB and incubated on surface-immobilized microtubules for 5 min. After incubation, 100 nM katanin–GFP was added to the measurement chamber in presence of the established tau concentration and the interaction was observed for 2 min. For control experiments, no tau was in the measurement chamber when katanin was introduced.

##### Tau-ΔN microtubule protection against katanin

Tau-ΔN or full-length phospho-tau was diluted in AB such that the tau density of tau-ΔN was similar to phospho-tau density outside envelopes (2 μM and 150 nM, respectively). Tau-ΔN or phospho-tau was incubated on microtubules for 5 min. After incubation, 350 nM katanin was added in the presence of established tau concentrations. For the control, no tau was added to the chamber. Katanin severing was observed for 5–10 min.

##### Elevated-pH treatment in vitro

Glycerol-stabilized GDP microtubules were immobilized on the coverslip surface and incubated in Gly40-AB (AB with BRB80 instead of HEPES–KCl and supplemented with 40% glycerol) pH 7.4 for 1 min before exchanging the medium for Gly40-AB pH 8.4 an incubated for an additional 9 min. As a control, Gly40-AB pH 7.4 was exchanged for new Gly40-AB pH 7.4.

##### Katanin assay on GMCPP-capped GDP microtubules

GMCPP-capped GDP microtubules were immobilized on the coverslip surface. Phospho-tau or dephospho-tau was diluted in AB such that the tau density was similar on GMPCPP and GDP lattices (120 nM or 30 nM, respectively) and incubated for 5 min. After incubation, 100 nM katanin was added while tau concentration remained constant and imaged until all microtubules were disassembled.

##### Katanin assay on GMPCPP and taxol-stabilized microtubules assay

GMPCPP-stabilized and taxol-stabilized microtubules were sequentially added to the measurement chamber and their position was identified using IRM. Phospho-tau–mCherry or dephospho-tau–mCherry was diluted in AB to concentrations at which the tau density on GMPCPP and taxol-stabilized microtubules was similar (120 nM and 30 nM, respectively). Tau was incubated on the microtubules for 5 min; then, 500 nM katanin was added in the presence of established tau concentration. Katanin severing was observed for 20 min.

##### Katanin on tau envelopes assay

Dephospho-tau–mCherry or phospho-tau–mCherry was diluted in AB and incubated on surface-immobilized microtubules for 5 min. Concentrations of dephospho-tau and phospho-tau were chosen to achieve similar envelope coverage (0.8 nM and 3.5 nM, respectively). After incubation, 100 nM katanin–GFP was added to the measurement chamber in presence of the established tau concentration. Katanin severing was observed for 30 min.

#### TIRF image analysis

Microscopy data were analyzed using ImageJ (version 2.3.0/1.53t; Fiji)^36^ and custom-written Matlab (versions R2020b and R2021a) codes. In images with substantial drift, the ‘StackregJ’ plugin was used to correct the drift (kindly provided by J. Unruh at Stowers Institute for Medical Research).

##### Kymographs

Kymographs were generated by drawing a line along the microtubule and using the ImageJ kymographBuilder plugin.

##### Envelope coverage

Microtubule lengths were measured using the IRM signal and tau envelope lengths were measured using the fluorescence signal after 3 min of incubation. Envelope boundaries were either established by eye or by using a linescan to define the envelope boundaries as the half-maximum of the signal. The envelope coverage represents the sum of all tau envelopes lengths divided by the sum of all microtubule lengths within one field of view.

##### Normalized coverage difference after Cdk5 treatment

Tau envelope coverage difference was calculated by subtracting the envelope coverage before the addition of active or deactivated Cdk5/p35 with the envelope coverage 15 min after adding active or deactivated Cdk5/p35 and normalized to the envelope coverage before addition.

##### Tau density estimation

Tau density (within or outside envelopes) on the microtubules was measured in ImageJ by drawing a rectangle around the microtubule and measuring the mean. For background subtraction, the rectangle was then moved to an area directly adjacent to the microtubule where no microtubule is present and the mean of the background was then subtracted from the mean on the microtubule.

##### Envelope disassembly rate because of Cdk5 treatment

The envelope length was measured before tau removal and after 5–45 min, as described above. The disassembly rate was calculated as the difference in envelope lengths before and after tau removal, divided by the time. If an envelope disappeared fully before the end of the video, the beginning length was divided by the time it took for the envelope to completely disassemble.

##### Envelope fissures because of Cdk5 treatment

Envelope fissures appearing during tau envelope disassembly were counted manually. The number of fissures in each envelope was then divided by the envelope length at the start of the video and by the time.

##### Hill coefficient

Tau density was measured along the entire length of the microtubules and plotted against the tau concentration added to the measurement chamber. The data were fitted with the Hill–Langmuir equation (*a* × *x*^*c*^)/(*b* + *x*^*c*^) using the curve-fitting tool in Matlab to obtain the Hill coefficient (*c*) and the *k*_D_ (*b*).

##### Tau-ΔN microtubule protection against katanin

Tau-ΔN and full-length phospho-tau density were measured and background-subtracted as described above. For the Tau-ΔN and control experiments, the density was measured along the entire microtubule. For the phospho-tau experiments, tau density was measured either within or outside the envelopes. Microtubule disassembly rate because of katanin severing was measured as described above.

##### Elevated-pH treatment in vitro

The length of the GDP microtubules was measured (1) before buffer exchange; (2) right after the exchange for buffer with pH 8.4 (or pH 7.4 in control); and (3) 9 min after the buffer exchange. The microtubule lengths at steps 2 and 3 were normalized to the length at step 1.

##### Normalized GMPCPP and GMPCPP-capped GDP microtubule disassembly

Katanin-mediated microtubule disassembly was measured by drawing a line along the microtubule in TIRF on either GMPCPP-capped GDP microtubules in GDP regions or GMPCPP microtubules and then measuring the mean intensity of tau signal within this region for each frame of the video. The background was subtracted as described above. Only microtubules with similar tau densities were included in the analysis. Data were normalized to the initial tau signal within each region.

##### Normalized GMPCPP and taxol-stabilized microtubule density

Katanin-mediated microtubule disassembly was measured by drawing a line along the microtubule in TIRF on either taxol-stabilized microtubules in enveloped regions or GMPCPP microtubules. The disassembly rate was measured as described above.

##### Katanin severing in tau envelope

Katanin severing events were counted manually from tau signal. Severing events were counted when a clear gap appeared in the tau–mCherry signal and when subsequent disassembly of the microtubule was observed from the newly acquired boundaries.

##### Normalized tau density in envelopes after katanin addition

Tau density was measured at five time points after the addition of katanin. The first frame after katanin addition was marked *t* = 0 min. Tau density was measured within the envelope region and normalized to the tau density within the same envelope at *t* = 0 min.

### Live-cell experiments

#### Plasmids

Human tau sequence (h441-tau) N-terminally tagged with eGFP or mCherry in pCDNA.4 vector was used as control tau. The tau sequence with a deleted N terminus (tau 242–441) was created from control tau using one-step site-directed deletion^37^ (primers: forward, CGGCCGCACGCCTGCAGACAGCCCCCGTGCCCAT; reverse, GCAGGCGTGCGGCCGCGGCTCCGAATTCTTTGTATAGT for eGFP-tagged control tau and GCAGGCGTGCGGCCGCGGCTCCGAATTCTAACTTGTA for mCherry-tagged control tau). The human tubulin sequence (TUBA1B) fused with mScarlet was used for lentiviral and retroviral particle production. To increase the phosphorylation level, cotransfection was used with vectors pCDNA3 overexpressing Cdk5 and p25. For katanin overexpression, cotransfection of pLL vectors expressing katanin subunit p60 and GFP-tagged katanin subunit p80 was used.

#### Tau lysate preparation

HEK293T cells, chosen for their high transfection efficiency, were cotransfected with GFP/mCherry-tagged tau and empty pCDNA3 vector (1:1) or with GFP/mCherry-tagged tau and pCDNA3 vectors overexpressing Cdk5 and p25 (1:0.85:0.15) using linear polyethylenimine (Polysciences). Cells were harvested 48 h after transfection by centrifugation and flash-frozen. Cell pellets were resuspended in 0.5 pellet volumes of lysis buffer (BRB80 supplemented with 1× phosphatase inhibitors (4906845001, Sigma), 1× protease inhibitors (04693159001, Sigma) and 0.05% Triton X-100 (Sigma)). The mixture was sonicated with three short pulses using the sonotrode MS1 (Hielscher Ultrasonics), setting cycle = 1 and amplitude = 100% (30 kHz) on ice. The solution was transferred to 270-µl Beckman ultracentrifuge tubes and ultracentrifuged in the Beckman 42.2 Ti rotor at 30,000*g*, 4 °C for 30 min in the Beckman Coulter Optima XPN-90 ultracentrifuge. The supernatant was directly used for experiments or flash-frozen in liquid nitrogen and stored at −80 °C. Tau concentrations in the lysates were measured at 488-nm absorbance using a NanoDrop ND-1000 spectrophotometer (Thermo Scientific).

#### Tau lysate imaging

Chambers were prepared as described above. Tau concentrations were estimated as described above and lysates were diluted in AB buffer to reach similar tau concentrations (about 30–40 nM tau−1GFP). Diluted tau lysates were then added to surface-immobilized taxol-stabilized microtubules where tau envelope formation was captured for 3 min. Envelope coverage was measured as described above.

#### Cell-cycle experiments

For the monitoring of tau during the cell cycle, we used U-2 OS cells for their well-established cell-cycle process. The U-2 OS human cell line (American Type Culture Collection, HTB-96) was transfected with the GFP–tau vector using the X-tremeGENE HP reagent (Sigma-Aldrich) and then selected with 200 µg ml^−1^ zeocin. GFP-positive cells were sorted by fluorescence-activated cell sorting (BD FACS Aria Fusion). Cells were then transduced with retroviral particles containing the mScarlet–tubulin. Briefly, platinum A cells were transfected with pMXs-Puro-mScarletI-tubulinα, particles were collected after 48 h and applied to cells, which were selected with 2.5 µg ml^−1^ puromycin. The resulting GFP–tau/mScarlet–tubulin cell line was grown on glass-bottom dishes in Fluorobrite medium with 10% FBS and glutamine and observed on a confocal Zeiss LSM 880 microscope at 37 °C and 5% CO_2_.

#### FRAP experiments

For live-cell imaging, we made use of IMCD-3 cells because of their large morphology and easily discernible and well-visible microtubule cytoskeleton. IMCD-3 cells were transduced by lentiviral particles produced by cotransfection of HEK293T cells with lentiviral vector carrying the sequence for mScarlet–tubulin together with gag/pol and vsv-g vector (1:0.9:0.1). Medium containing lentiviral particles was collected 48 h after transfection, filtered (0.45-µm pores) and used for transduction of IMCD-3 cells.

IMCD-3 cells expressing mScarlet–tubulin were then transfected in OptiMEM media (Thermo Scientific) using Lipofectamine 2000 (Thermo Scientific) according to the manufacturer’s protocol. Cells were transfected in eight-well chambered coverslips (Ibidi) using 0.5 µg of DNA per well. Cotransfection of GFP–tau, Cdk5 and p25 was performed in the ratio 1:0.85:0.15. The cells were grown in DMEM/F12 supplemented by FBS and penicillin–streptomycin (Thermo Scientific).

A spinning-disk confocal microscope (Nikon CSU-W1) equipped with an FRAP and photoactivation module was used to image FRAP cells. Cells were imaged using a CF Plan Apo VC 60XC WI objective (water immersion), 488-nm laser and FITC filter. Imaging was performed on a single cell using three different settings: (1) the cell was imaged for 17 frames (100-ms exposure time, 500-ms interval) before FRAP; (2) FRAP was performed on a circular region of 0.5-μm diameter; (3) the cell was imaged for 22 s directly after FRAP to visualize the recovery. Imaging was performed at 37 °C and 5% CO_2_.

#### Elevated-pH treatment and live-cell imaging

For live-cell imaging, IMCD-3 cells were prepared as described above. Cells were imaged every 20 s for 10 min. After 1 min of imaging, the medium (DMEM/F12 supplemented with FBS and penicillin–streptomycin) was changed to regular medium with pH adjusted to 8.4 with NaOH. Cells were imaged in TIRF mode (Apo TIRF ×60 oil DIC N2; 488 + 561 nm; exposure: 300 ms) using OKO-lab chamber (37 °C, 5% CO_2_).

#### Katanin experiment in cells (preparation)

IMCD-3 cells were cotransfected with vectors for overexpression of mCherry–tau or mCherry, katanin subunit p60, katanin subunit p80–GFP, and Cdk5 and p25 or empty pCDNA3 vector (in the ratio: 1: 0.375: 0.375: 0.375: 0.375). Alternatively, IMCD-3 cells were cotransfected with vectors for overexpression of mCherry–tau or mCherry–tau-∆N (N-terminally truncated tau), katanin subunit p60, and katanin subunit p80–GFP (in the ratio: 1: 0.375: 0.375). For the analysis of Cdk5/p25 effect on microtubules, IMCD-3 cells were cotransfected with vectors for overexpression of mCherry and Cdk5 and p25 or pcDNA3 empty vector (in the ratio 1:0.375:0.375). 12 hours after transfection the cells were fixed using 4% PFA/PBS for 15 min followed by methanol at -20 °C for 2 min. Fixed cells were kept in PBS at 4 °C.

#### Katanin experiment in cells (immunostaining)

IMCD-3 cells were blocked for 1 h in 0.1% BSA in PBS and then stained with anti-β-tubulin antibody overnight (1:400; DSHB Hybridoma Product E7 deposited to the Developmental Studies Hybridoma Bank^38^). After washing, cells were incubated with anti-mouse secondary antibody conjugated with Alexa-647 (Thermo Scientific). Cells were captured using a confocal microscope Leica Stellaris 8 (HC PL APO CS2 ×63/1.40 oil, white-light laser).

For analysis of the effect of Cdk5/p25 on microtubules, anti-β-tubulin and anti-p35 primary antibodies and anti-mouse or anti-rabbit secondary antibody conjugated with Alexa Fluor 647 or 488 (1:400, Thermo Scientific), respectively, were used.

### Live-cell image analysis

#### Coefficient of variation

To determine the coefficient of variation (CoV) for monitoring GFP–tau during the cell cycle, whole cells were manually selected. For the elevated-pH treatment experiments, a circle was drawn inside the cell, covering (most of) the area of the cell (in the case of large cells, two circular areas were averaged). The CoV was determined using ImageJ by measuring the s.d. of the tau fluorescence signal within the region of interest (ROI), divided by the mean.

#### Pearson’s R

Cells in interphase or in mitosis were manually selected on the basis of the presence or absence of a mitotic spindle and Pearson’s correlation coefficient between the GFP–tau and the mScarlet–tubulin channels was calculated with the Coloc 2 plugin in ImageJ.

#### FRAP analysis (recovery time constant)

The intensity of the GFP signal was measured in (1) a small ROI on a microtubule in the bleached region of the cell; (2) the same-size region on a unbleached microtubule in the same cell, used as a reference; and (3) the same-size region outside the cell, used as the background. In total, 14–15 cells were analyzed. The curves were double-normalized according to the following equation:

*F*_FRAP-normalized_(*t*) = (*F*_ref-pre_/(*F*_ref_(*t*) − *F*_bg_(*t*)))((*F*_FRAP_(*t*) − *F*_bg_(*t*)]/*F*_FRAP-pre_), where *F*_ref-pre_ = Σ_(*t* = 0;*t* = 17)_ ((*F*_ref_(*t*) *−* *F*_bg_(*t*))/*F*_prebleach_), *F*_FRAP-pre_ = Σ_(*t* = 0;*t* = 17)_ ((*F*_FRAP_(*t*) *−* *F*_bg_(*t*))/*F*_prebleach_), *F*_prebleach_ = 17, *F*_ref_(*t*) is the reference fluorescence intensity on the microtubule in the same cell but not in the bleached region, *F*_FRAP_(*t*) is the fluorescence intensity on the microtubule in the bleached ROI, *F*_bg_(*t*) is the fluorescence intensity in a background ROI outside the cells, *F*_ref-pre_ is the mean fluorescence intensity of the reference ROI before the bleaching after background subtraction and *F*_FRAP-pre_ is the mean fluorescence intensity of the bleached ROI before the bleaching after background subtraction. The normalized data were fitted using the Matlab fitting tool using the equation: *y* = *a* × exp(−*b* *×* *x*) + *c*, where *b* is the rate constant and *c* is the asymptote.

#### FRAP analysis (immobile fraction)

The percentage of immobile fraction was measured using the equation (1 − (*c* − *F*_0_)/(1 − *F*_0_)) × 100, where *c* is the asymptote (of the fitted curve) and *F*_0_ is the normalized intensity immediately after bleaching.

#### Normalized microtubule length difference (elevated-pH treatment)

Total microtubule and tau envelope lengths were measured within selected ROIs (0.01 mm^2^) using ImageJ. First, a mask covering the tau envelopes or microtubules within the area was generated using intensity thresholding and tubeness plugin. Subsequently, the total length of microtubules or tau envelopes was quantified from the mask using the skeletonize plugin. The effect of the pH treatment was calculated as a change in microtubule (or tau envelope) length before and directly after (within 20–40 s) the pH treatment. In total, 13 randomly selected ROIs within seven cells from four independent experiments were quantified.

#### Tau density on microtubules (elevated-pH treatment)

The mean intensity of tau on the microtubule was measured before elevated-pH treatment and divided by the mean intensity of the same-size region in the cytoplasm next to the measured microtubule at the same time point. The average of five microtubules was used for each cell.

#### Mean intensity of tau in the cell (elevated-pH treatment)

The mean intensity of the GFP signal was measured in random circular regions of the cytoplasm before elevated-pH treatment.

#### Normalized tau density in patches (elevated-pH treatment)

The intensity of tau in the patches (visible in tau signal) at five different time points after elevated-pH treatment was analyzed. For tau-∆N, the intensity over the entire length of the microtubule was analyzed. The mean intensity in a tau-positive region on the microtubule (tau patch or full microtubule) was measured and subtracted by the mean intensity of the same-size region next to the microtubule. All time points were normalized to the intensity of tau before the elevated-pH treatment.

#### Normalized tau density on microtubules (elevated-pH treatment)

The mean tau intensity in the cell (ROI comprising most of the cell) was measured at all time points and subtracted by the intensity of the GFP signal in the cytoplasm next to the microtubules. The intensity at each time point was then normalized to the intensity before elevated-pH treatment. Both curves (control tau cells and tau-Cdk5 cells) were fitted using Matlab fitting tool using the exponential recovery curve: f(*x*) = *a* − *b* × exp(−*c* × *x*) with exponential time constant 1/*c* (min).

#### Katanin experiment (relative tubulin and katanin density)

The mean density of stained tubulin signal in the transfected cell relative to the mean density of surrounding cells nontransfected with katanin was analyzed using ImageJ (average tubulin intensity of three circular ROIs in the cytoplasm of the transfected cell divided by the average tubulin intensity in three circular ROIs in three randomly selected nontransfected cells in close vicinity to the analyzed cell). The relative tubulin density was either correlated to the relative density of the katanin signal (correlation plot with the linear regression) or plotted according to the experimental groups in a scatter plot (for this purpose, only cells with a relative intensity of katanin of 0.5–6 were plotted in the tau-Cdk5 figure and relative intensities of katanin of 0–200 for the tau-∆N figure). The data were then fitted using Matlab fitting tool using the linear curve: f(*x*) = *a* × *x* + 1.

#### Katanin experiment (relative tau density in cells)

The mean density of tau signal in the transfected cells was analyzed using ImageJ (average tau intensity of three circular ROIs in the cytoplasm of the transfected cell subtracted by the average background intensity in three circular ROIs). The values were normalized to the average intensity of the control group (+tau).

### MS

Samples were analyzed using a liquid chromatography (LC) system Agilent 1200 (Agilent Technologies) connected to the timsToF Pro parallel accumulation–serial fragmentation (PASEF) MS instrument equipped with Captive spray (Bruker Daltonics). The MS instrument was operated in a positive data-dependent mode. First, 5 μl of peptide mixture was injected by an autosampler on the C18 trap column (ultrahigh-performance LC fully porous polar C18, inner diameter: 2.1 mm; Phenomenex). After 5 min of trapping at a flow rate of 20 µl min^−1^, peptides were eluted from the trap column and separated on a C18 column (Luna Omega, 3 μm, polar C18, 100 Å, 150 × 0.3 mm; Phenomenex) by a linear 35-min water–acetonitrile gradient from 5% (v/v) to 35% (v/v) acetonitrile at a flow rate of 4 µl min^−1^. The trap and analytical columns were both heated to 50 °C. Parameters from the standard proteomics PASEF method were used to set timsTOF Pro. The target intensity per individual PASEF precursor was set to 6,000 and the intensity threshold was set to 1,500. The scan range was set between 0.6 and 1.6 V s cm^−2^ with a ramp time of 100 ms. A total of ten PASEF MS/MS scans were performed. Precursor ions in the *m*/*z* range between 100 and 1,700 with charge states ≥2+ and ≤6+ were selected for fragmentation. The active exclusion was enabled for 0.4 min. The raw data were processed by PeaksStudio 10.0 software (Bioinformatics Solutions). The search parameters were set as follows: enzyme trypsin (specific), carbamidomethylation as a fixed modification and oxidation of methionine, phosphorylation (STY) and acetylation of protein N terminus as variable modifications.

### MS sample preparation

#### Tau samples

Tau with different levels of phosphorylation (phospho-tau, dephospho-tau, Bact-tau and Bact-tau-Cdk5) was prepared as described above.

#### Spun down samples

First, 1 μM phospho-tau or 200 nM Bact-tau-Cdk5 was diluted in MS buffer (50 mM HEPES, 75 mM KCl, 10 µM taxol, 10 mM dithiothreitol and 1 mM Mg-ATP) and added to taxol-stabilized microtubules. Microtubules and tau were incubated for 10 min at room temp and centrifuged for 30 min at 18,000*g* in a Microfuge 18 centrifuge (Beckman Coulter). After centrifugation the supernatant was separated from the pellet and used as the sample indicated by ‘tau in solution’. The pellet was resuspended in a fourfold lower volume of MS buffer and centrifuged again to ensure a more homogeneous sample. After the second centrifugation, the supernatant was discarded and the pellet was resuspended in BRB80 before being used as the sample indicated by ‘tau in envelopes‘.

### MS analysis

#### Phosphorylation degree

For each phosphorylation site, all peptides were studied that include the specific site, phosphorylated or not. Each peptide is found with a relative intensity, which gives an indication of the density at which that peptide is detected in the sample. The sum of the relative intensities of the peptide in phosphorylated state was divided by the sum of the total relative intensities of the peptide (nonphosphorylated + phosphorylated) to obtain the phosphorylation degree of the phosphorylation site. Each tau sample was prepared in triplicate and each independent sample was analyzed separately, resulting in three individual phosphorylation degree values for each phosphorylation site. The graphs display the mean ± s.d. for each sample at each phosphorylation site, plotted along the amino acid sequence of tau or as individual phosphorylation sites.

#### Total relative intensity

For each phosphorylation site, all relative intensities at which the peptides covering the specific phosphorylation were summed to get the total relative intensity for the specific phosphorylation site.

### Western blot experiments

HEK tau lysates, insect tau and bacterial tau samples were prepared as stated above. Samples were boiled in sample buffer (50 mM TrisCl pH 6.8, 2% SDS, 2% β-mercaptoethanol, 10% glycerol and 0.1% bromophenol blue) at 98 °C for 10 min. Equal amounts of tau were separated on 7.5% SDS–PAGE gel and proteins were then transferred onto PVDF membrane. Membranes were blocked using 5% milk in TBS–Tween and probed with primary antibodies. After incubation with horseradish peroxidase (HRP)-conjugated secondary antibody, the signal was developed using enhanced chemiluminescence and visualized on Chemidoc (Bio-Rad). The following antibodies were used: tau5 (1:3,000; Santa Cruz), AT8 (1:3,000; Thermo Fisher Scientific), AT180 (1:3,000; Thermo Fisher Scientific), phospho-tau S404 (1:1,000; Thermo Fisher Scientific) and secondary HRP-conjugated anti-mouse or anti-rabbit antibody (1:10,000; Life Technologies). Densitometric quantification of protein bands was performed using ImageJ. The intensity of phosphorylated tau was normalized to the corresponding total tau intensity.

### Statistics and reproducibility

For representative plots and figures, whenever not specifically stated in the caption, all data were collected from at least three independent trials. All repeated independent experiments showed similar results and no data were excluded from the manuscript. Unless stated otherwise, all data were analyzed manually using ImageJ (Fiji), Matlab (version R2020b or R2021a) or QuPath (version 0.5.1). Graphs were created using Matlab or Prism 10 and statistical analyses were performed using the same software. Major points on graphs represent data means and error bars represent the s.d., unless stated otherwise.

### Reporting summary

Further information on research design is available in the Nature Portfolio Reporting Summary linked to this article.

## Online content

Any methods, additional references, Nature Portfolio reporting summaries, source data, extended data, supplementary information, acknowledgements, peer review information; details of author contributions and competing interests; and statements of data and code availability are available at 10.1038/s41589-025-02122-9.

## Supplementary information

Supplementary Information Supplementary video legends.

Reporting Summary

Supplementary Video 1 Related to Fig. 1c: 1.5 nM phospho-tau on taxol-stabilized microtubules. A time-lapse movie of 1.5 nM phospho-tau–GFP (magenta) added to surface-immobilized taxol-stabilized microtubules and imaged for 5 min.

Supplementary Video 2 Related to Fig. 1c: 1.5 nM dephospho-tau on taxol-stabilized microtubules. A time-lapse movie of 1.5 nM dephospho-tau–GFP (cyan) added to surface-immobilized taxol-stabilized microtubules and imaged for 5 min.

Supplementary Video 3 Related to Fig. 1e: 10 nM Bact-tau in the presence of active Cdk5. A time-lapse movie of 10 nM Bact-tau–GFP (magenta) on surface-immobilized taxol-stabilized microtubules (black). Active Cdk5/p35 was added to the measurement chamber at *t* = 0 min.

Supplementary Video 4 Related to Fig. 1e: 10 nM Bact-tau in the presence of deactivated Cdk5. A time-lapse movie of 10 nM Bact-tau–GFP (cyan) on surface-immobilized taxol-stabilized microtubules (black). Deactivated Cdk5/p35 (control) was added to the measurement chamber at *t* = 0 min.

Supplementary Video 5 Related to Fig. 2f: 10 nM Bact-tau removal in the presence of active Cdk5. A time-lapse movie of 10 nM Bact-tau–GFP (magenta) on surface-immobilized taxol-stabilized microtubules (black). Bact-tau was removed from solution in the presence of active Cdk5/p35.

Supplementary Video 6 Related to Fig. 2f: 10 nM Bact-tau removal in the presence of deactivated Cdk5. A time-lapse movie of 10 nM Bact-tau–GFP (cyan) on surface-immobilized taxol-stabilized microtubules (black). Bact-tau was removed from solution in the presence of deactivated Cdk5/p35.

Supplementary Video 7 Related to Fig. 3a: FRAP of tau–GFP (control) cell. A time-lapse movie showing an IMCD-3 cell overexpressing GFP–tau (control; cyan) on which FRAP was performed at *t* = 0 min. The GFP–tau signal was monitored for 10 s before FRAP and 20 s after FRAP.

Supplementary Video 8 Related to Fig. 3a: FRAP of tau-∆N–GFP cell. A time-lapse movie showing an IMCD-3 cell overexpressing GFP–tau-∆N (tau-∆N) on which FRAP was performed at *t* = 0 min. The GFP–tau signal was monitored for 10 s before FRAP and 20 s after FRAP.

Supplementary Video 9 Related to Fig. 3a: FRAP of tau-Cdk5–GFP cell. A time-lapse movie showing an IMCD-3 cell overexpressing GFP–tau-Cdk5/p25 (tau-Cdk5) on which FRAP was performed at *t* = 0 min. The GFP–tau signal was monitored for 10 s before FRAP and 20 s after FRAP.

Supplementary Video 10 Related to Extended Data Fig. 6a: pH treatment on tau (control) cell. A time-lapse movie showing an IMCD-3 cell overexpressing GFP–tau (control) on which elevated-pH treatment was performed at *t* = 0 min. The GFP–tau signal (left) and mScarlet–tubulin signal (right) were monitored for 10 min: 1 min before treatment and 9 min after treatment.

Supplementary Video 11 Related to Extended Data Fig. 6a: pH treatment on tau-∆N cell. A time-lapse movie showing an IMCD-3 cell overexpressing GFP–tau-∆N (tau-∆N) on which elevated-pH treatment was performed at *t* = 0 min. The GFP–tau signal (left) and mScarlet–tubulin signal (right) were monitored for 10 min: 1 min before treatment and 9 min after treatment.

Supplementary Video 12 Related to Extended Data Fig. 6a: pH treatment on tau-Cdk5 cell. A time-lapse movie showing an IMCD-3 cell overexpressing GFP–tau-Cdk5/p25 (tau-Cdk5) on which elevated-pH treatment was performed at *t* = 0 min. The GFP–tau signal (left) and mScarlet–tubulin signal (right) were monitored for 10 min: 1 min before treatment and 9 min after treatment.

Supplementary Video 13 Related to Fig. 5a: Katanin severing GMPCPP microtubules while GMPCPP-capped GDP microtubules are protected by phospho-tau. A time-lapse movie showing katanin–GFP (yellow) added at *t* = 0 min severing phosho-tau-covered GMPCPP microtubules, while GMPCPP-capped GDP microtubules covered by the same density of phospho-tau (magenta) are protected.

Supplementary Video 14 Related to Extended Data Fig 8a: Katanin severing GMPCPP microtubules while GMPCPP-capped GDP microtubules are protected by dephospho-tau. A time-lapse movie showing katanin–GFP (yellow) added at *t* = 0 min severing dephospho-tau-covered GMPCPP microtubules, while GMPCPP-capped GDP microtubules covered by the same density of dephospho-tau (cyan) are protected.

Supplementary Video 15 Related to Fig. 5d: Katanin severing GMPCPP microtubules while taxol-stabilized microtubules are protected by phospho-tau. A time-lapse movie showing katanin–GFP (yellow) added at *t* = 0 min severing phosho-tau-covered GMPCPP microtubules, while taxol-stabilized microtubules covered by the same density of phospho-tau (magenta) are protected.

Supplementary Video 16 Related to Fig. 5d: Katanin severing GMPCPP microtubules while taxol-stabilized microtubules are protected by dephospho-tau. A time-lapse movie showing katanin–GFP (yellow) added at *t* = 0 min severing dephosho-tau-covered GMPCPP microtubules, while taxol-stabilized microtubules covered by the same density of dephospho-tau (cyan) are protected.

## Source data

Source Data Figs. 1–6 Statistical source data.

Source Data Extended Data Figs. 1–10 Statistical source data.

Source Data Extended Data Fig. 1 Unprocessed western blots.

Source Data Extended Data Fig. 2 Unprocessed western blots.

Source Data Extended Data Fig. 4 Unprocessed western blots.

## Data Availability

Additional data for all figures are available with this paper or Supplementary Information. Source data are provided with this paper.
