# Supplementary material for: Tau phosphorylation impedes functionality of protective tau envelopes
Source: Nat Chem Biol. 2026 Jan 27;22(5):759–69. doi: 10.1038/s41589-025-02122-9 (PMC13128492; doi:10.1038/s41589-025-02122-9)
Supplement: Supplementary file 1 — Supplementary video legends. [file 41589_2025_2122_MOESM1_ESM.pdf]

nature chemical biology

Supplementary information

https://doi.org/10.1038/s41589-025-02122-9

# Tau phosphorylation impedes functionality of protective tau envelopes

In the format provided by the authors and unedited

## Supporting information

### Supplementary movie legends

**Movie 1** related to Fig. 1c: **1.5nM phospho-tau on taxol-stabilized microtubules.** A time-lapse movie of 1.5 nM phospho-tau-GFP (magenta) added to surface-immobilized taxol-stabilized microtubules and imaged for 5 minutes.

**Movie 2** related to Fig. 1c: **1.5nM dephospho-tau on taxol-stabilized microtubules.** A time-lapse movie of 1.5 nM dephospho-tau-GFP (cyan) added to surface-immobilized taxol-stabilized microtubules and imaged for 5 minutes.

**Movie 3** related to Fig. 1e: **10nM Bact-tau in presence of active Cdk5.** A time-lapse movie of 10 nM Bact-tau-GFP (magenta) on surface-immobilized taxol-stabilized microtubules (black). Active Cdk5/p35 was added to the measurement chamber at t=0 min.

**Movie 4** related to Fig. 1e: **10nM Bact-tau in presence of deactivated Cdk5.** A time-lapse movie of 10 nM Bact-tau-GFP (cyan) on surface-immobilized taxol-stabilized microtubules (black). Heat-deactivated Cdk5/p35 (control) was added to the measurement chamber at t=0 min.

**Movie 5** related to Fig. 2f: **10nM Bact-tau removal in presence of active Cdk5.** A time-lapse movie of 10 nM Bact-tau-GFP (magenta) on surface-immobilized taxol-stabilized microtubules (black). Bact-tau was removed from solution in presence of active Cdk5/p35.

**Movie 6** related to Fig. 2f: **10nM Bact-tau removal in presence of deactivated Cdk5.** A time-lapse movie of 10 nM Bact-tau-GFP (cyan) on surface-immobilized taxol-stabilized microtubules (black). Bact-tau was removed from solution in presence of heat-deactivated Cdk5/p35.

**Movie 7** related to Fig. 3a: **FRAP of tau-GFP (control) cell.** A time-lapse movie showing an IMCD-3 cell overexpressing GFP-tau (control, cyan) on which FRAP is performed at t=0 sec. GFP-tau signal was monitored for 10 seconds before FRAP and 20 seconds after FRAP.

**Movie 8** related to Fig. 3a: **FRAP of tau-GFP-deltaN cell.** A time-lapse movie showing an IMCD-3 cell overexpressing GFP-tau-ΔN (tau-ΔN) on which FRAP is performed at t=0 sec. GFP-tau signal was monitored for 10 seconds before FRAP and 20 seconds after FRAP.

**Movie 9** related to Fig. 3a: **FRAP of tau-GFP-Cdk5 cell.** A time-lapse movie showing an IMCD-3 cell overexpressing GFP-tau in combination with Cdk5/p25 (tau-Cdk5) on which FRAP is performed at t=0 sec. GFP-tau signal was monitored for 10 seconds before FRAP and 20 seconds after FRAP.

**Movie 10** related to Extended Data Fig. 6a: **pH treatment on tau (control) cell.** A time-lapse movie showing an IMCD-3 cell overexpressing mScarlet-tubulin and GFP-tau (control) on which elevated-pH treatment is performed at t=0 min. GFP-tau signal (left) and mScarlet-tubulin signal (right) were monitored for 10 minutes; 1 min before treatment and 9 minutes after treatment.

**Movie 11** related to Extended Data Fig. 6a: **pH treatment on tau-ΔN cell.** A time-lapse movie showing an IMCD-3 cell overexpressing mScarlet-tubulin and GFP-tau-ΔN (tau-ΔN) on which elevated-pH treatment is performed at t=0 min. GFP-tau signal (left) and mScarlet-tubulin signal (right) were monitored for 10 minutes; 1 min before treatment and 9 minutes after treatment.

**Movie 12** related to Extended Data Fig. 6a: **pH treatment on tau-Cdk5 cell.** A time-lapse movie showing an IMCD-3 cell overexpressing mScarlet-tubulin and GFP-tau in combination with Cdk5/p25 (tau-Cdk5) on which elevated-pH treatment is performed at t=0 min. GFP-tau signal (left) and mScarlet-tubulin signal (right) were monitored for 10 minutes; 1 min before treatment and 9 minutes after treatment.

**Movie 13** related to Fig. 5a: **Katanin severing GMPCPP microtubules while GMPCPP-capped GDP microtubules are protected by phospho-tau.** A time-lapse movie showing katanin-GFP (yellow) added at t=0min severing phosho-tau (magenta) covered GMPCPP microtubules, while GMPCPP-capped GDP microtubules covered by the same density of phospho-tau are protected.

**Movie 14** related to Extended Data Fig. 8a: **Katanin severing GMPCPP microtubules while GMPCPP-capped GDP microtubules are protected by dephospho-tau.** A time-lapse movie showing katanin-GFP (yellow) added at t=0min severing dephospho-tau (cyan) covered GMPCPP microtubules, while GMPCPP-capped GDP microtubules covered by the same density of dephospho-tau are protected.

**Movie 15** related to Fig. 5d: **Katanin severing GMPCPP microtubules while taxol-stabilized microtubules are protected by phospho-tau.** A time-lapse movie showing katanin-GFP (yellow) added at t=0min severing phosho-tau (magenta) covered GMPCPP microtubules, while taxol-stabilized microtubules covered by the same density of phospho-tau are protected.

**Movie 16** related to Fig. 5d: **Katanin severing GMPCPP microtubules while taxol-stabilized microtubules are protected by dephospho-tau.** A time-lapse movie showing katanin-GFP (yellow) added at t=0min severing dephosho-tau (cyan) covered GMPCPP microtubules, while taxol-stabilized microtubules covered by the same density of dephospho-tau are protected.
