# Supplementary material for: Tau phosphorylation impedes functionality of protective tau envelopes
Source: Nat Chem Biol. 2026 Jan 27;22(5):759–69. doi: 10.1038/s41589-025-02122-9 (PMC13128492; doi:10.1038/s41589-025-02122-9)
Supplement: Supplementary file 2 — Reporting Summary [file 41589_2025_2122_MOESM2_ESM.pdf]

nature portfolio

Corresponding author(s): Zdenek Lansky
Martin Balastik
Marcus Braun

Last updated by author(s): Nov 18, 2025

Reporting Summary

Nature Portfolio wishes to improve the reproducibility of the work that we publish. This form provides structure for consistency and transparency in reporting. For further information on Nature Portfolio policies, see our Editorial Policies and the Editorial Policy Checklist.

Statistics

For all statistical analyses, confirm that the following items are present in the figure legend, table legend, main text, or Methods section.

n/a	Confirmed	
☐	☒	The exact sample size (*n*) for each experimental group/condition, given as a discrete number and unit of measurement
☐	☒	A statement on whether measurements were taken from distinct samples or whether the same sample was measured repeatedly
☐	☒	The statistical test(s) used AND whether they are one- or two-sided<br>*Only common tests should be described solely by name; describe more complex techniques in the Methods section.*
☒	☐	A description of all covariates tested
☒	☐	A description of any assumptions or corrections, such as tests of normality and adjustment for multiple comparisons
☐	☒	A full description of the statistical parameters including central tendency (e.g. means) or other basic estimates (e.g. regression coefficient) AND variation (e.g. standard deviation) or associated estimates of uncertainty (e.g. confidence intervals)
☐	☒	For null hypothesis testing, the test statistic (e.g. *F*, *t*, *r*) with confidence intervals, effect sizes, degrees of freedom and *P* value noted<br>*Give P values as exact values whenever suitable.*
☒	☐	For Bayesian analysis, information on the choice of priors and Markov chain Monte Carlo settings
☒	☐	For hierarchical and complex designs, identification of the appropriate level for tests and full reporting of outcomes
☐	☒	Estimates of effect sizes (e.g. Cohen's *d*, Pearson's *r*), indicating how they were calculated

Our web collection on statistics for biologists contains articles on many of the points above.

Software and code

Policy information about availability of computer code

Data collection	Data were collected using Nikon NIS Elements; Leica Application Suite X (LAS X); Bruker Compass HyStar 6.0
Data analysis	Data were analyzed using FIJI 2.3.0/1.53t (ImageJ); Matlab (R2020b, R2021a); PEAKS Studio 10.0 (Bioinformatics Solutions); QuPath 0.5.1.

For manuscripts utilizing custom algorithms or software that are central to the research but not yet described in published literature, software must be made available to editors and reviewers. We strongly encourage code deposition in a community repository (e.g. GitHub). See the Nature Portfolio guidelines for submitting code & software for further information.

Data

Policy information about availability of data

All manuscripts must include a data availability statement. This statement should provide the following information, where applicable:

- Accession codes, unique identifiers, or web links for publicly available datasets
- A description of any restrictions on data availability
- For clinical datasets or third party data, please ensure that the statement adheres to our policy

Source data files for all figures and all extended data figures are available with the manuscript. A statement on data availability is included in the manuscript.

Research involving human participants, their data, or biological material

Policy information about studies with human participants or human data. See also policy information about sex, gender (identity/presentation), and sexual orientation and race, ethnicity and racism.

Reporting on sex and gender	n/a
Reporting on race, ethnicity, or other socially relevant groupings	n/a
Population characteristics	n/a
Recruitment	n/a
Ethics oversight	n/a

Note that full information on the approval of the study protocol must also be provided in the manuscript.

Field-specific reporting

Please select the one below that is the best fit for your research. If you are not sure, read the appropriate sections before making your selection.

☒ Life sciences ☐ Behavioural & social sciences ☐ Ecological, evolutionary & environmental sciences

For a reference copy of the document with all sections, see nature.com/documents/nr-reporting-summary-flat.pdf

Life sciences study design

All studies must disclose on these points even when the disclosure is negative.

Sample size	Sample sizes were chosen within the consensus in the field; meaning that during each independent experiment (i.e. in each individual flow channel or in each culture dish or well), microscopy fields of view were randomly chosen. In each field of view, the number of molecules, microtubules, or cells determined the sample size per measurement. If experimentally possible, several fields of view per flow channel/well were imaged, increasing the sample size and randomizing the experiment. Sample sizes are limited by experimental feasibility.
Data exclusions	No data were excluded from the manuscript.
Replication	Experiments were performed over several months by several experimentalists. All replication attempts (that were not impeded by unrelated events, like image acquisition software malfunction) were successful. For each quantified experiment and each exemplary image or kymograph, 'n' describes the number of biologically individual samples (individual molecules, microtubules, cells, etc.). Details are given in the figure legends.
Randomization	Biological samples (proteins, microtubules) as well as other assay components were taken randomly in small volumes by pipetting from stock solutions of larger volumes and allocated randomly to the different experimental groups. During experiments, random fields of view were chosen. For analysis either all events in a field of view or all events on a random filament or in a random cell were chosen and analyzed.
Blinding	Investigators were necessarily blinded to group allocation as it is impossible to influence or select any sub-population of biological samples (proteins, filaments or any other assay components) that are taken from the stock solutions during pipetting.

Reporting for specific materials, systems and methods

We require information from authors about some types of materials, experimental systems and methods used in many studies. Here, indicate whether each material, system or method listed is relevant to your study. If you are not sure if a list item applies to your research, read the appropriate section before selecting a response.

Materials & experimental systems

n/a	Involved in the study
☐	☒ Antibodies
☐	☒ Eukaryotic cell lines
☒	☐ Palaeontology and archaeology
☒	☐ Animals and other organisms
☒	☐ Clinical data
☒	☐ Dual use research of concern
☒	☐ Plants

Methods

n/a	Involved in the study
☒	☐ ChIP-seq
☒	☐ Flow cytometry
☒	☐ MRI-based neuroimaging

Antibodies

Antibodies used

anti-β-tubulin antibodies (#T7816, Sigma); anti-biotin antibodies (#B3640, Sigma); anti-β-tubulin antibodies E7 (DSHB, antibody registry ID: AB_2315513); anti-p35 antibody (sc-820, Santa Cruz); Anti-Tau5 (sc-58860, Santa Cruz); anti-phospho tau Ser202, Thr205 AT8 (MN1020, Thermofisher Scientific), anti-phospho tau Thr231 AT180 (MN1040, Thermofisher Scientific); anti-phospho tau Ser404 (44-758G, Thermofisher Scientific); Goat anti-Mouse IgG secondary antibody with Alexa Fluor 647 (A-21236, Thermofisher Scientific); Goat anti-Rabbit IgG secondary antibody with Alexa Fluor 647 (A-21245, Thermofisher Scientific)

Validation

The anti-β-tubulin (Sigma) and anti-biotin antibodies (Sigma) were used to immobilize microtubules on the coverslip surface for all in vitro experiments. The microtubule sample was purified and the biotin-labeled tubulin was bought (#T333P, Cytoskeleton Inc.), meaning that only tubulin was present in the sample. The fact that the microtubules bound to the antibody-coated surface thus validates the use of these antibodies for this application. Goat anti-Mouse and anti-Rabbit IgG (1:400; ThermoFisher Scientific) are commercial fluorescent secondary antibodies. Their specificity was tested by immunocytochemistry without primary antibodies with no signal detected. Anti-β-tubulin, (1:400; E7; DSHB) used for in vivo katanin experiments, are well-characterized widely used monoclonal antibodies. Their specificity was confirmed by immunocytochemistry in IMCD-3 cell line, where characteristic microtubule pattern and their loss upon katanin (microtubule severing enzyme) treatment were specifically detected, thereby validating the use of this antibody. To test co-transfection efficiency, the anti-p25 antibody (1:1000 for WB, 1:300 for IF; sc-820, Santa Cruz) was used and validated by Western blot using neuronal lysates, where a 35 kDa band corresponding to endogenous p35 was detected, and a 25 kDa band was observed in lysates from neurons transfected with p25, confirming the antibody's ability to recognize both isoforms. Immunofluorescence in IMCD-3 cells transfected with p25 detected signal only in transfected cells, further supporting antibody specificity. For Western Blots, the anti-tau antibodies Tau5 (1:3000; Santa Cruz) and anti-phospho tau antibodies AT8, AT180, and phospho-S404 (1:3000, 1:3000 and 1:1000, respectively; all from Thermofisher Scientific) were validated by Western blot using lysates containing recombinant or overexpressed tau. All antibodies detected bands at the expected size. The phospho-specific antibodies showed reduced signal after lysate dephosphorylation and increased signal with kinase upregulation, confirming phosphorylation-dependent specificity.

Eukaryotic cell lines

Policy information about cell lines and Sex and Gender in Research

Cell line source(s)

IMCD-3 cell line was purchased from ATCC (CRL-2123); HEK293T cell line was obtained from collaborator Meritxell Alberich Jorda, Ph.D. (Institute of Molecular Genetics, Czech Academy of Sciences); U-2 OS human cell line was purchased from ATCC (HTB-96)

Authentication

Commercial cells were not authenticated.

Mycoplasma contamination

Cell cultures were routinely tested for Mycoplasma contamination using the Mycoplasma Detection Kit from ATCC (#90-1001K) or the MycoAlert PLUS Mycoplasma Detection Kit (Lonza, LT07-710). All tests were negative.

Commonly misidentified lines
(See ICLAC register)

No commonly misidentified cell lines were used in this study.
