# Supplementary material for: Tau phosphorylation impedes functionality of protective tau envelopes
Source: Nat Chem Biol. 2026 Jan 27;22(5):759–69. doi: 10.1038/s41589-025-02122-9 (PMC13128492; doi:10.1038/s41589-025-02122-9)
Supplement: Supplementary file 21 — Unprocessed western blots. [file 41589_2025_2122_MOESM21_ESM.pdf]

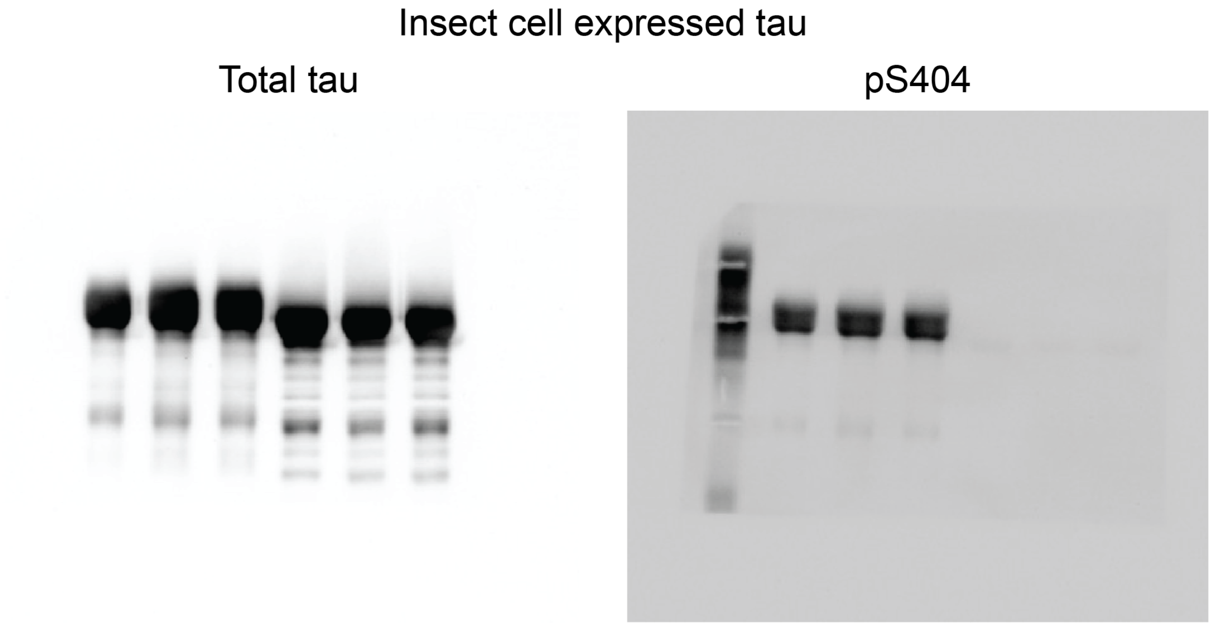

Uncropped images for Extended data Fig. 1c

Western blot of insect cell expressed tau (first 3 lanes) and phosphatase-treated insect cell expressed tau (last 3 lanes) using total tau antibody (tau-5, left panel) and phospho-specific antibody (pS404, right panel).
