# Supplementary material for: Tau phosphorylation impedes functionality of protective tau envelopes
Source: Nat Chem Biol. 2026 Jan 27;22(5):759–69. doi: 10.1038/s41589-025-02122-9 (PMC13128492; doi:10.1038/s41589-025-02122-9)
Supplement: Supplementary file 23 — Unprocessed western blots. [file 41589_2025_2122_MOESM23_ESM.pdf]

HEK293T cell lysates

Total tau

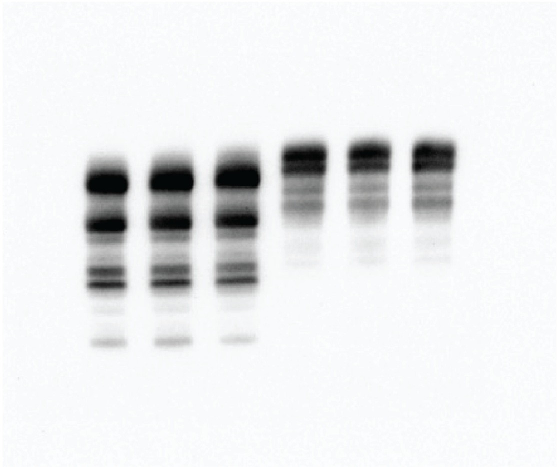

AT8

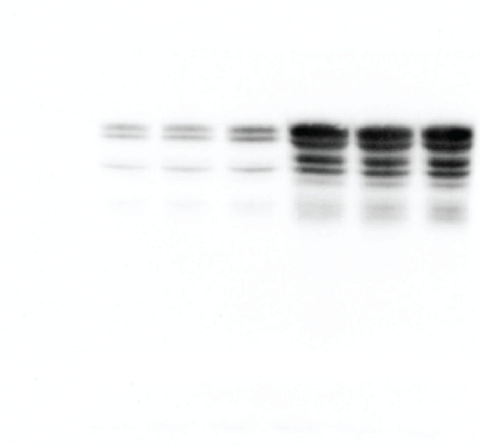

AT180

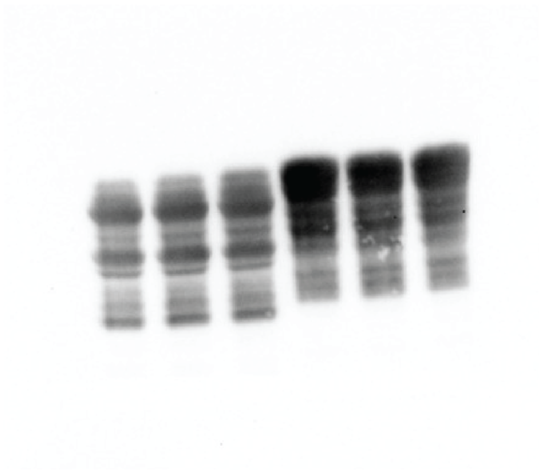

pS404

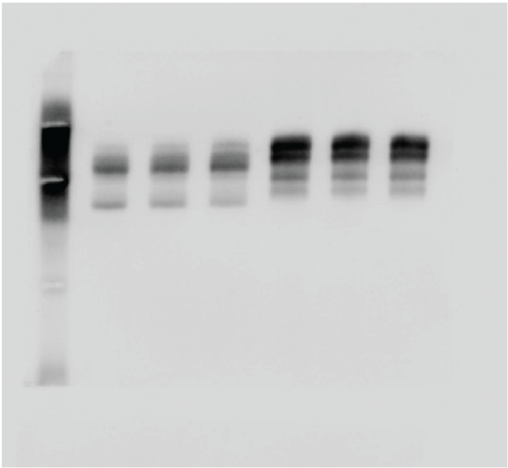

Uncropped images for Extended Data Fig. 4b

Western blot of HEK293T cells transfected with GFP-tau (first 3 lanes) and cells transfected with GFP-tau and Cdk5/p25 (last 3 lanes) using total tau antibody (tau-5, top panel) and phospho-specific antibodies (AT8, AT180 and pS404).
